# Antisense oligonucleotide modulation of non-productive alternative splicing upregulates gene expression

**DOI:** 10.1038/s41467-020-17093-9

**Published:** 2020-07-09

**Authors:** Kian Huat Lim, Zhou Han, Hyun Yong Jeon, Jacob Kach, Enxuan Jing, Sebastien Weyn-Vanhentenryck, Mikaela Downs, Anna Corrionero, Raymond Oh, Juergen Scharner, Aditya Venkatesh, Sophina Ji, Gene Liau, Barry Ticho, Huw Nash, Isabel Aznarez

**Affiliations:** Stoke Therapeutics, Inc., Bedford, MA USA

**Keywords:** Antisense oligonucleotide therapy, RNA splicing

## Abstract

While most monogenic diseases are caused by loss or reduction of protein function, the need for technologies that can selectively increase levels of protein in native tissues remains. Here we demonstrate that antisense-mediated modulation of pre-mRNA splicing can increase endogenous expression of full-length protein by preventing naturally occurring non-productive alternative splicing and promoting generation of productive mRNA. Bioinformatics analysis of RNA sequencing data identifies non-productive splicing events in 7,757 protein-coding human genes, of which 1,246 are disease-associated. Antisense oligonucleotides targeting multiple types of non-productive splicing events lead to increases in productive mRNA and protein in a dose-dependent manner in vitro. Moreover, intracerebroventricular injection of two antisense oligonucleotides in wild-type mice leads to a dose-dependent increase in productive mRNA and protein in the brain. The targeting of natural non-productive alternative splicing to upregulate expression from wild-type or hypomorphic alleles provides a unique approach to treating genetic diseases.

## Abstract (web summary)

Restoration of normal gene expression is one way to treat monogenic disorders. Here the authors target naturally occurring non-productive alternative splicing using antisense oligonucleotides to promote the production of functional proteins.

## Introduction

Over 10,000 monogenic diseases have been identified that affect millions of individuals worldwide (https://www.who.int/). Monogenic disorders are the result of a single defective gene and inheritance patterns can be autosomal dominant (one mutant allele), recessive (both alleles are mutant), or X-linked. Autosomal dominant diseases are further divided into haploinsufficiency (the mutant allele leads to loss of function) or dominant-negative/gain-of-function (the mutant allele leads directly to a toxic function). Current therapeutic approaches address as little as 5% of these diseases and the majority are only addressing patient symptoms^1,2^. Most monogenic disorders are caused by reduced expression or deficient proteins. Efforts to restore the level or function of missing or altered proteins have led to the development of gene therapy, CRISPR, modified RNA, recombinant protein, and antisense approaches. However, these approaches have various limitations and therefore are not suitable to treat many genetic diseases, especially haploinsufficiency^3–9^. As it is difficult to restore normal gene expression levels in a tissue-specific and titratable manner, there remains a significant need for alternative therapeutic approaches.

In eukaryotic cells, precursor messenger RNAs (pre-mRNAs) can be extensively modified by a process termed pre-mRNA splicing^10^. During splicing, introns are removed and exons are joined together to generate the mRNA template that carries the code to synthesize proteins. It is estimated that more than 90% of mammalian genes are alternatively spliced to generate multiple mRNAs from a single gene, thereby increasing proteomic diversity in multicellular organisms^11^. More than a third of these alternative splicing (AS) events in mammals are non-productive, as they lead to the introduction of a premature termination codon (PTC) and subsequent degradation through the nonsense-mediated mRNA decay (NMD) pathway^12^. NMD is an evolutionarily conserved RNA surveillance system initially thought to selectively mitigate deleterious effects of PTC-containing mRNA transcripts as a result of disease mutations^13^. More recent studies have demonstrated that NMD also regulates the overall abundance of productive transcripts by coupling AS with NMD^14^. In fact, a striking number of splicing factors autoregulate their own expression through this coupling mechanism^15,16^. As the expression of a broad class of physiological transcripts can be downregulated by AS-NMD^17^, we reasoned that antisense-oligonucleotide (ASO)-mediated modulation of splicing can be applied to prevent non-productive (NMD-inducing) AS events, thereby increasing productive mRNA and protein levels in a gene-specific manner.

As a therapeutic modality, ASOs take advantage of Watson & Crick-base pairing to target RNA processing and modulate protein expression with high specificity. ASOs are generally 15–30 nucleotides in length and contain chemical modifications in the sugar as well as the backbone, which increase stability (nuclease resistant), prevent immune response, and increase binding affinity^8^. Applications of single-stranded ASOs include the downregulation of gene expression by eliciting RNaseH-mediated degradation of the target transcript (gapmers) and the prevention of binding of proteins to RNA (steric-blocking). Steric blocking ASOs modulate splicing by preventing splicing factors from binding to the target pre-mRNA. In turn this approach can address mutations that cause splicing defects, avoid deleterious mutations, or switch protein isoforms^18^. In studies on β-thalassemia and spinal muscular atrophy, steric-blocking ASOs were used to rescue splicing defects caused by specific mutations by promoting inclusion of coding exons, leading to fully functional proteins^19,20^. In contrast, to avoid specific deleterious nonsense or frameshift mutations in a mouse model for Duchenne muscular dystrophy ASOs were utilized to cause skipping of the coding exons where these mutations reside, resulting in partially functional truncated proteins^21,22^. To date, ASOs have only been used to address specific mutations that affect a subset of patients with monogenic diseases. One exception is the mutation that affects the splicing of survival of motor neuron 2 (*SMN2*), which is a backup copy gene that is almost identical to *SMN1* and can make up for the absence of *SMN1* expression^20^. This scenario appears to be unique to the *SMN* genes. There remains an unmet need for therapeutic approaches using ASOs that can address an entire patient population of haploinsufficient diseases in a mutation-independent manner.

Only two ASO chemistries are utilized in currently marketed ASO drugs approved by the U.S. Food & Drug Administration (FDA) and they are phosphorodiamidate morpholino oligomer (PMO) and 2′ methoxyethyl (2′MOE)^8^. In this study, we utilize PMO and 2′MOE to design a therapeutic approach that could address patients with monogenic loss-of-function diseases. Our approach, termed targeted augmentation of nuclear gene output (TANGO), exploits ASO-mediated modulation of naturally occurring non-productive AS events. We show that TANGO can increase full-length, fully functional protein expression by leveraging wild-type alleles. Furthermore, the upregulation is not limited by the size of the gene and the effect is titratable.

## Results

### Identification and validation of NMD-inducing AS events

To identify non-productive AS events in organs known to be accessible by ASOs, 83 publicly available RNA-sequencing (RNA-seq) datasets from human liver^23^, kidney^24^, central nervous system (CNS)^25^, and eye tissues^26^ were analyzed. To ensure data quality, several parameters were used to assess both raw and mapped sequencing reads suggested by Conesa and colleagues^27^, including the overall sequence quality score, average percentage of guanine-cytosine (GC) contents, the presence of adapters, duplicated reads, and overrepresented sequences. After alignment, a minimum of 70% capture efficiency was required by measuring the percentage of total sequenced reads uniquely mapped to the transcriptome. Coverage uniformity and the distribution of insert size were also evaluated. Subsequently, 5 of the 83 samples that did not satisfy these quality control measures were removed from further analyses (Supplementary Data 1).

**Fig. 1:**
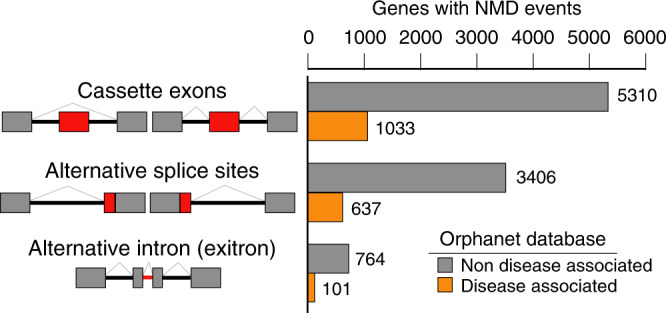
Identification of protein-coding genes with NMD-inducing events. 7757 unique genes containing at least one non-productive AS events, of which 1246 unique genes are disease-associated. The left panel depicts the types of AS events (cassette exons, alternative splice sites, and alternative intron/exitron) that are predicted to undergo NMD. Exons are denoted by rectangles and introns by lines. Red rectangles or red lines denote non-productive AS region. Gray rectangles denote protein-coding exons. The right panel horizontal bar graph summarizes the number of protein-coding genes containing each of the non-productive AS event type. Gray bars depict non-disease-associated genes and orange bars depict disease-associated genes (source: Orphanet disease database). Note: genes can have multiple NMD events of different types (Supplementary Data 2), therefore some genes are included in more than one type of event. To calculate the total number of unique genes (7757 and 1246 unique disease-associated genes) with non-productive events, each gene was counted only once. Source data are provided as a Source Data file.

The abundance of NMD-sensitive transcripts is typically low since they are actively degraded in the analyzed tissues. To identify these events, requirements were set at a minimum of three read pairs uniquely mapped to splice junctions and a minimum of 3% of non-productive splicing (calculated as percent spliced-in index, PSI, and relative to protein-coding splicing). Splice junctions were defined as non-productive if the AS event produced a stop codon at least 50 nucleotides upstream of the 3′ most exon–exon junction. With these thresholds, our computational analysis discovered 7757 unique genes containing at least one non-productive AS event. By cross-referencing these events with genetic disease databases such as Orphanet (https://www.orpha.net/), a total of 1246 potentially disease-associated genes with at least one non-productive AS events were identified (Fig. 1, Supplementary Data 2).

To prove the TANGO concept broadly, target selection was based on several criteria. First, non-productive AS events were grouped into the following categories: alternative cassette exon, alternative splice sites, and alternative intron (exitron) (Fig. 1, Supplementary Data 2). These in-silico predicted NMD events were validated in cells where NMD can be inhibited and the “true” abundance of the non-productive events can be assessed. To this end, cells were treated with cycloheximide (CHX), a translation inhibitor commonly used to inhibit NMD^28^. After 3 h of treatment with 50 μg/ml of CHX or equal volume of DMSO (control), cells were harvested and RNA was extracted. Using this approach, 70% (1985 out of 2831) of the events assayed were positively validated in cells. We speculate that the 30% discrepancy could be due to using different sources of RNA for the bioinformatics analysis (tissues) and the validation experiments (cells). When the bioinformatics predictions and validations were performed using the same source, 94% of the events assayed were positively validated.

Supplemental Fig. 1 shows examples of RT-PCR validation results for 14 genes with non-productive AS events representative of each event type (cassette exon, alternative splice sites, and alternative intron; Supplemental Data 3). Percentage of non-productive splicing (non-productive over non-productive + productive) was calculated via densitometric analysis of PCR products. Results show a consistent increase in the predicted non-productive splicing upon CHX treatment compared to control DMSO-treated cells (Supplementary Fig. 1a–n), as expected for AS events that lead to transcript degradation by NMD. The observed increase in abundance after NMD inhibition compared to DMSO control suggests that the NMD efficiency is different across all non-productive events (~1.1- to 9-fold increase). The resulting post-NMD inhibition abundance of the non-productive AS events relative to total (productive + non-productive) ranges from 18.6% (*MRGBP*) to 89% (*SCN1A*) in cells (Supplementary Fig. 1a–n). These results indicate that our bioinformatics pipeline is capable of identifying different types of non-productive AS events of variable abundance that can be leveraged to increase protein expression.

In addition, the genes were further classified into different categories to evaluate whether protein upregulation is possible regardless of the size of coding sequence (CDS), protein type, or function. As upregulation of gene expression could be beneficial for a variety of diseases, it was pertinent to determine whether or not the genes are disease-associated. Using these criteria, four genes were selected for further experimental testing among the validated targets shown in Supplemental Fig. 1 (Table 1 and Supplementary Fig. 1c, f, l, m). Collectively, these four genes represent three types of non-productive AS events with a wide range of abundance, small to large CDS, and four different types of protein functions (metabolic enzyme, post-synaptic neuron signaling pathway, immune response signaling pathway, and voltage-gated sodium channel).

**Table 1:** Candidate genes for TANGO proof of concept.

Gene	AS type	NMD^a^ (%)	CDS^b^ (bp)	Protein	Disease
*PCCA*	Cassette exon	30	2184	PCCA – metabolic enzyme	Propionic acidemia (AR)
*SYNGAP1*	Alternative splice site	47	4029	SynGAP – signaling pathway/synapse	AD mental retardation 5 (haploinsufficiency)
*CD274*	Alternative intron (exitron)	20	870	PD-L1 – signaling pathway/immune response	Disease-relevant pathway (eye/autoimmune uveitis)
*SCN1A*	Cassette exon	89	6015	Na_V_ 1.1 – ion channel	Dravet syndrome (AD) (haploinsufficiency)

*AR* autosomal recessive, *AD* autosomal dominant.

^a^Post-NMD inhibition (see Supplementary Fig. 1c, f, l, m).

^b^CDS: coding sequence (from start to stop codon).

### Systematic ASO walks targeting non-productive AS events

**Fig. 2:**
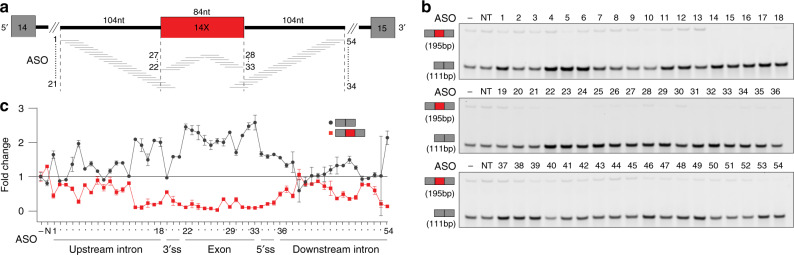
ASO walk targeting the NMD-inducing exon inclusion in *PCCA*. **a** Diagram of ASO walk. The target region spans 104 nt of intronic sequences on each side of the non-productive alternative exon 14X, the exon–intron boundaries and exon 14X. Black lines denote introns, gray rectangles denote constitutive exons, and red rectangle denotes non-productive alternative exon. **b** A representative RT-PCR PAGE from HEK293 cells transfected with a total of 54 18-mer ASOs (shown in panel **a**) and a non-targeting ASO control (NT) at 80 nM concentration or no-ASO mock control (−) for 24 h. The top band (195 bp) corresponds to the non-productive mRNA that results from the inclusion of exon 14X and the bottom band (111 bp) corresponds to the productive mRNA that results from the skipping of exon 14X (*n* = 2 biologically independent samples over two independent experiments). **c** Graph depicting the fold increase over no-ASO mock control (−) of *PCCA* productive mRNA determined by TaqMan qPCR (gray circles, *n* = 2) and the fold decrease of *PCCA* non-productive mRNA based on densitometric quantification of RT-PCR products (red squares, *n* = 2) from panel **b**. Data are presented as mean values ±SD. Results show an inverse correlation between the reduction of non-productive mRNA and the increase of productive mRNA. ASO sequences are in Supplementary Data 4. Source data are provided as a Source Data file.

To identify ASOs that can prevent the non-productive AS events and potentially increase gene expression, an initial systematic ASO walk in 5-nt steps was performed along the AS event of interest. All ASOs used in these initial walks are 18-nt long and have a uniform phosphorothioate backbone and methoxyethyl at the 2′ ribose position (2′MOE-PS) (Supplementary Data 4). These modifications were previously shown to bind RNA with high affinity and to confer resistance to both nucleases and RNase H cleavage of the target RNA^29^. Studies have demonstrated successful targeting of splicing mutations^30^ with efficacious clinical outcomes^8^ to warrant the selection of this chemistry. We selected *PCCA* from the two non-productive cassette exon events as an example of an ASO walk to prevent the inclusion of a non-productive exon. To this end, we designed a 54-ASO walk to target exon 14X in *PCCA* and its adjacent intronic flanks (Fig. 2a). HEK 293 cells were transfected with 80 nM of each *PCCA* targeting ASO and a non-targeting (NT) control in addition to a no-ASO (−) mock control. RT-PCR analysis designed to detect both non-productive and productive splicing identified several ASOs that reduce exon 14X inclusion in the *PCCA* mRNA and increase productive mRNA by splicing together exons 14 and 15 (Fig. 2b, e.g. ASOs 22–33). The intensities of the PCR products were measured by densitometry and used to calculate the percentage of the non-productive splicing as described above. The fold changes of exon 14X inclusion were plotted relative to the no-ASO mock control (RT-PCR) (Fig. 2c, red curve). Furthermore, the increase in *PCCA* productive mRNA was confirmed by TaqMan qPCR using a probe that spans the spliced junction of exons 14 and 15. The expression of the productive mRNA measured by qPCR is first normalized to an endogenous housekeeping gene, then to the no-ASO control and plotted as fold change (Fig. 2c, gray curve). The results show an inverse relationship between the decrease in non-productive splicing (RT-PCR) and the increase in productive mRNA (qPCR), suggesting that the reduction of non-productive splicing event caused by ASOs can lead to mRNA upregulation.

**Fig. 3:**
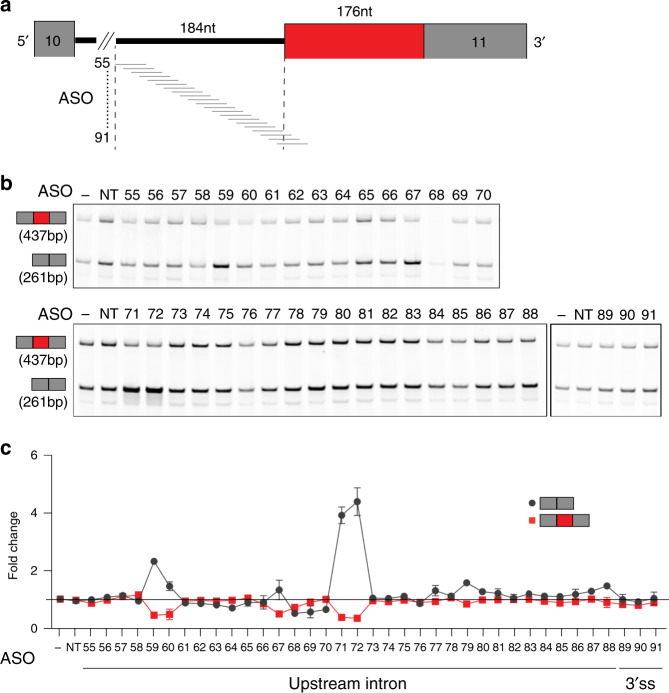
ASO walk targeting the NMD-inducing alternative 3′ss in *SYNGAP1*. **a** Diagram of ASO walk. The target region spans 184 nt of intronic sequences upstream as well as across the non-productive alternative 3′ss in *SYNGAP1*. Black lines denote introns, gray rectangles denote constitutive exons, and red rectangle denotes non-productive alternative region. **b** A representative RT-PCR PAGE from HEK293 cells transfected with a total of 37 18-mer ASOs (shown in panel **a**) and a non-targeting ASO control (NT) at 80 nM concentration or no ASO control (−) for 24 h. The top band (437 bp) corresponds to the non-productive mRNA that results from the alternative 3′ss and the bottom band (261 bp) corresponds to the productive mRNA that results from the canonical splicing of exon 11 (*n* = 2 biologically independent samples over two independent experiments). **c** Graph depicting the fold decrease of *SYNGAP1* non-productive mRNA based on densitometric quantification of RT-PCR products (red squares, *n* = 2) from panel **b** and the fold increase over no ASO control (−) of *SYNGAP1* productive mRNA determined by TaqMan qPCR (gray circles, *n* = 2). Data are presented as mean values ±SD. Results show an inverse correlation between the reduction of non-productive mRNA and the increase of productive mRNA. ASO sequences are in Supplementary Data 4. Source data are provided as a Source Data file.

To determine whether other types of non-productive splicing events can be leveraged to increase gene expression, two additional non-productive AS types were targeted: an NMD-inducing alternative 3′ splicing event in *SYNGAP1* and an NMD-inducing alternative intron (exitron) in *CD274*. As described above, 18-mer 2′MOE-PS ASOs were designed to target the alternative 3′ splice site (3′ss) region (Fig. 3a) and the alternative intron region (Supplementary Fig. 2a) in 5-nt steps. The corresponding *SYNGAP1* and *CD274* targeting ASOs were transfected into HEK293 cells and Huh7 cells, respectively. As the *CD274* non-productive alternative intron event (AI) is efficiently degraded (Supplementary Fig. 1f), Huh7 cells were treated with CHX for 3 h prior to harvesting to ensure accurate measurement of the non-productive mRNA. RT-PCR analysis of *SYNGAP1* identified five functional ASOs that reduce the use of the non-productive alternative 3′ss and increase the productive mRNA by promoting splicing of exons 10 and 11 (Fig. 3b, ASO 59, 60, 67, 71, 72). The upregulation of *SYNGAP1* productive mRNA was also confirmed by TaqMan qPCR (Fig. 3b, gray curve). Furthermore, several ASOs were identified that decrease *CD274* non-productive alternative intron removal (Supplementary Fig. 2b, c, red curve) and increase the productive mRNA (Supplementary Fig. 2c, gray curve). Similar to the mechanism of action of the *PCCA* targeting ASOs, we observed an inverse relationship between the reduction of the non-productive mRNA and the increase of the productive mRNA for both *SYNGAP1* and *CD274* (Fig. 3c and Supplementary Fig. 2c, respectively). These results demonstrate that the ASOs in this study upregulate gene expression on mechanism by leveraging non-productive AS events.

To determine the role that length and chemical modifications of the identified hit ASOs has on their ability to modulate splicing, 20–23 mers were designed to be centered in the region encompassing *SYNGAP1* ASOs 59 and 60 using 2′MOE-PS modifications as well as PMO. RT-PCR and TaqMan qPCR analyses of nucleofected cells show that both 2′MOE ASOs and PMOs can engage the target and increase productive mRNA (Supplemental Fig. 3a–c, respectively). These results suggest that ASOs of variable lengths designed with two difference chemical modifications function via the same mechanism of action as the parental 18-mer 2′MOE-PS hits.

### Dose-dependent upregulation of mRNA and protein in vitro

**Fig. 4:**
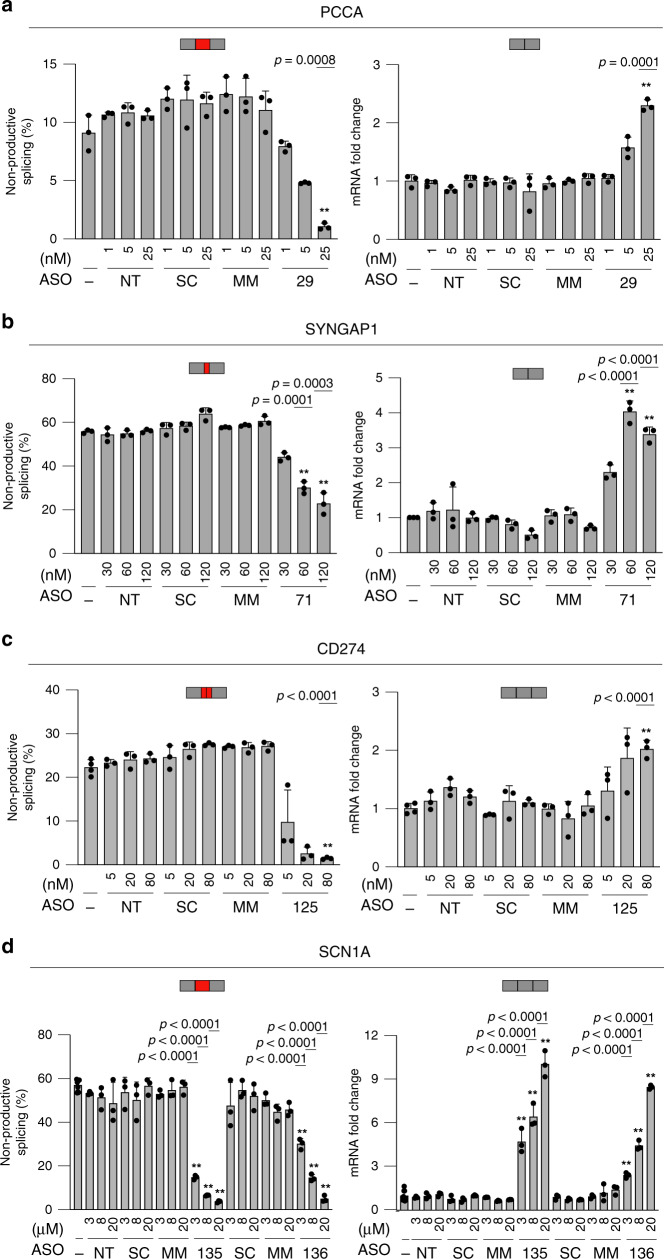
ASOs dose-dependently reduce NMD and increase mRNA in vitro. **a** RT-PCR (left panel) and qPCR (right panel) of a selected *PCCA* ASO (ASO-29) targeting the exon inclusion event transfected in HEK293 cells at increasing concentrations for 24 h. Exact *p*-values for bars with asterisks are 0.0008 and 0.0001, respectively. **b** RT-PCR (left panel) and qPCR (right panel) of a selected *SYNGAP1* ASO (ASO-71) targeting the alternative 3′ss transfected in HEK293 cells at increasing concentrations for 24 h. Exact *p*-values for bars with asterisks are 0.0001 and 0.0003, 6.99e-5 and 4.25e-5, respectively. **c** RT-PCR (left panel) and qPCR (right panel) of a selected *CD274* ASO (ASO-125) targeting the alternative intron transfected in Huh7 cells at increasing concentrations for 24 h. Cell were treated with 50 μg/mL of CHX for 3 h prior to harvesting to visualize and quantify the non-productive mRNA. Exact *p*-values for bars with asterisks are 6.38e-6 and 9.21e-5, respectively. **d** RT-PCR (left panel) and qPCR (right panel) of two selected *SCN1A* ASOs (ASO-135 and ASO-136) targeting the exon inclusion event delivered by free uptake into ReNCell VM cells at increasing concentrations for 72 h. RT-PCR results (bar graphs on the left) show dose-dependent reductions of the non-productive mRNA and qPCR results (bar graphs on the right) show dose-dependent increases of productive mRNA. Exact p-values for bars with asterisks are 1.69e-10, 2.50e-11, and 1.49e-11; 2.91e-8, 2.05e-10, and 2.59e-11; 4.21e-7, 1.10e-8, and 1.35e-10; and 7.26e-5, 4.01e-8, and 9.08e-12, respectively. No-ASO (−), scramble (SC), and mismatch (MM) controls were included in each experiment at the same increasing concentrations as the respective targeting ASOs. All experiments were performed in three biological replicates. Data are presented as mean values ±SD. *P*-values were calculated using two-sided *t*-test. Asterisks denote ASOs that are statistically significant (*p* < 0.001) in both RT-PCR and qPCR analyses. Red and gray rectangles denote NMD-inducing event and protein-coding exons, respectively. Source data are provided as a Source Data file.

To determine whether the extent of ASO-mediated upregulation can be titrated, positive ASO hits from the initial walks were selected to perform dose-response experiments. To this end, cells were treated with positive hits (*PCCA* ASO-29, *SYNGAP1* ASO-71, *CD274* ASO-125, and *SCN1A* ASO-135 & 136) at increasing concentrations to demonstrate dose-dependent upregulation. The concentration range for each hit was determined based on the potency of each ASO and delivery method. To ensure meaningful interpretation and to show specificity of the selected ASOs, non-targeting (NT), and corresponding scramble (SC), and mismatch (MM, six mismatches) ASO controls were included in each experiment at the same increasing concentrations as the respective targeting ASOs. RT-PCR analyses of HEK293 cells transfected with ASO-29 and ASO-71 result in a dose-dependent decrease of the non-productive exon inclusion (EI) level in *PCCA* (Fig. 4a, left panel, Supplementary Fig. 4a) and the non-productive alternative 3′ss selection in *SYNGAP1* (Fig. 4b, left panel, Supplementary Fig. 4b), respectively. In both cases, the dose-dependent reduction of the non-productive AS events led to a dose-dependent increase of *PCCA* and *SYNGAP1* productive mRNAs measured by qPCR, compared to mock (−) control (Fig. 4a, b, right panels). None of the control ASOs (NT, SC, and MM) had any meaningful effects on splicing or gene upregulation of the respective targets. Similarly, Huh7 cells were transfected with the *CD274* hit ASO (ASO-125) at increasing doses, as well as a mock control, NT control, and its corresponding scrambled and mismatched ASO controls. In order to properly quantify target engagement of ASO-125, Huh7 cells were treated with CHX for 3 h. RT-PCR analysis shows a dose-dependent decrease of the *CD274* non-productive splicing (Fig. 4c, left panel, Supplementary Fig. 4d). The dose-dependent-reduction of the non-productive alternative intron led to a dose-dependent increase in productive mRNA measured by qPCR compared to mock control (Fig. 4c, right panel). The corresponding ASO controls (NT, SC, and MM) did not alter splicing or upregulate *CD274* transcript levels. In addition, two positive hits were selected from an ASO walk designed to target the inclusion of the non-productive exon 20X in *SCN1A*. Gymnotic (free) uptake of increasing concentrations of ASO-135 and ASO-136 in ReNcell VM (neural progenitor cells) led to a dose-dependent decrease of the non-productive exon (Fig. 4d left panel, Supplementary Fig. 4e). As expected, reducing the inclusion of the non-productive exon results in an inversely correlated increase in productive mRNA measured by qPCR compared to mock control (Fig. 4d, right panel). As with the previous examples, none of the ASO controls affected splicing or upregulated *SCN1A* expression.

**Fig. 5:**
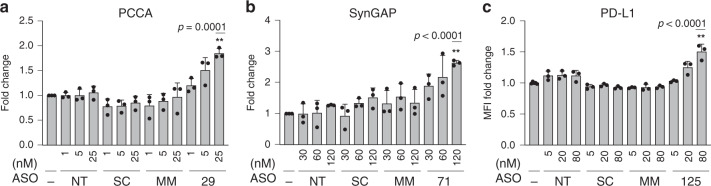
ASOs increase protein levels in a dose-dependent manner in vitro. **a** Bar graph plotting the quantification of PCCA western blot analysis from HEK293 cells treated with hit ASO at increasing concentrations for 48 h. Equal protein loading was confirmed with Ponceau staining (Supplementary Fig. 5). **b** Same for SynGAP (*p* = 4.62e-6). **c** Bar graph plotting flow cytometry derived fold change of the Mean Fluorescent Intensity of PD-L1 from Huh7 cells treated with the hit ASO at increasing concentrations for 120 h. No-ASO (−), scramble (SC), and mismatch (MM) controls were included in each experiment at the same increasing concentrations as the respective targeting ASOs (*p* = 7.70e-5). All experiments were performed in three biological replicates. Data are presented as mean values ±SD. *P*-values were calculated based on two-sided *t*-test. Asterisks indicate *p* < 0.001. Source data are provided as a Source Data file.

To determine whether the observed upregulation in productive mRNAs translates to a dose-dependent increase in protein levels, PCCA, SynGAP, and PD-L1 proteins (Na_V_ 1.1 protein levels were not quantifiable in ReNcell VM) were measured. To this end, protein extracts were prepared from transfected cells with increasing concentrations of targeting ASOs, as well as a non-targeting control, and corresponding scramble and mismatch controls. First, antibodies against PCCA and SynGAP were validated by short interfering (si)RNA-mediated knockdown of protein expression and western blot analysis (Supplementary Fig. 5a, b), while a commonly used antibody against PD-L1 protein^31,32^ was validated by overexpressing PD-L1 and performing flow cytometry (Supplementary Fig. 5c). Immunoblotting results of extracts from HEK 293 cells transfected with ASOs showed dose-dependent increases in PCCA (Fig. 5a, Supplementary Fig. 6a) and SynGAP (Fig. 5b, Supplementary Fig. 6b) proteins. Protein normalization using either Ponceau or vinculin, an internal control protein, led to similar results (Supplemental Fig. 7a–f). Likewise, PD-L1 protein levels in ASO-treated Huh-7 cells measured by flow cytometry show a dose-dependent increase of membrane-bound PD-L1 (Fig. 5c; Supplementary Fig. 6c).

Overall, the average level of target protein upregulation resulting from the highest ASO concentrations ranged between 1.5- and 2.6-fold. These results indicate that upregulation of target productive mRNAs leads to an increase of the respective proteins. The observed protein increase correlates with the level of productive mRNA increase, such that the ranking for productive mRNA upregulation (*CD274* – lower, *PCCA* – middle, and *SYNGAP1* – higher) is the same as the protein upregulation ranking (PD-L1 – lower, PCCA – middle, and SynGAP – higher). Moreover, the effect of the ASO hits is titratable, which allows for a tight control over the level of ASO-mediated upregulation.

Protein upregulation was also assessed using a 23-mer PMO (ASO-144) targeting *SYNGAP1* alternative 3′ss event. Immunoblotting results of nucleofected cells show similar level of protein upregulation as the parental ASO-59 (Supplemental Fig. 3d, e). These results suggest that the observed upregulation of productive mRNA and protein is translated from the initial 2′MOE-PS chemistry to PMO.

### Dose-dependent upregulation of mRNA and protein in vivo

In order to prove the concept of the TANGO technology in vivo, a sequence conservation analysis was first performed to select non-productive splicing events that are conserved between human and mouse. To this end, the analysis was limited to non-productive EI events (non-coding) to avoid any sequence conservation bias due to coding regions (exon skipping (ES), exitrons, exonic alternative 3′ss or 5′ss) or splice site regions (intronic alternative 3′ss and 5′ss). Results from the conservation analysis suggests that ~16% of the non-productive EI events are as conserved at the sequence level as coding exons (Supplementary Fig. 8a, calculated based on conservation scores ≥0.8). This observation is consistent with previous studies that have shown that non-productive (poison exons) alternative splicing events in RNA-binding proteins are highly conserved at the sequence level^33,34^. Here, we found that these highly conserved non-productive splicing events are also present in other types of genes, suggesting that AS-NMD regulates the abundance of a wide variety of transcripts. One example is the *SCN1A* non-productive AS event^35^ in which the non-coding exon and flanking intronic sequences are 96% conserved between human and mouse (Supplementary Fig. 8b). To confirm that the non-productive exon is also alternatively spliced in mouse cells, Neuro 2a (N2a) cells were treated with CHX as described above and RT-PCR analysis was performed to measure the level of the non-productive EI (human exon 20x corresponds to mouse exon 21x). Results showed that exon 21x is alternatively spliced in mouse cells (Supplementary Fig. 8c) and its inclusion leads to transcript degradation as was observed in human cells (Supplementary Fig. 1m). The observed conservation on both genomic sequence and splicing allowed us to test the human targeting ASOs in mice without any sequence mismatch and to ascertain whether the observed effect of the ASOs in vitro can be recapitulated in vivo.

**Fig. 6:**
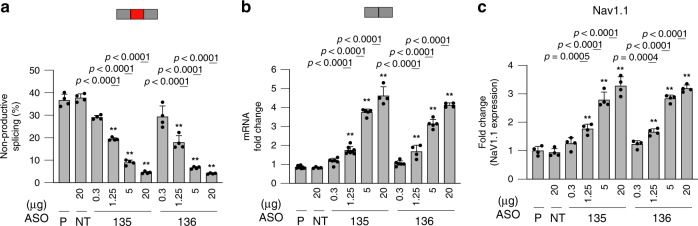
ASOs increase *Scn1a* mRNA and Na_v_1.1 protein expression in vivo. WT C57BL/6N mice (males and females) were ICV-injected at postnatal 2 with PBS (P), a non-targeting ASO control (NT), and two selected ASOs (135 and 136, human ASOs with perfect match to the mouse sequence) targeting the non-productive exon inclusion in *Scn1a*. **a** Percentage of NMD-inducing exon 21x inclusion in *Scn1a* transcript from mouse brains as quantified by densitometry of RT-PCR products (*n* = 4 randomly selected samples) (Supplementary Fig. 7). **b** Quantification of *Scn1a* productive transcript expression by probe-based qPCR (*n* = 9, PBS; *n* = 4, NT; *n* = 6, 8, 5, and 4, increasing doses of ASO-135, respectively; *n* = 6, 4, 5, and 4, increasing doses of ASO-136, respectively. **c** Quantification of Na_v_1.1 protein expression by MSD (*n* = 4 randomly selected samples). Red and gray rectangles denote NMD-inducing and protein-coding exons, respectively. Data are presented as mean values ±SD. *P*-values were calculated based on two-sided *t*-test. Asterisks denote ASOs that are statistically significant (*p* < 0.001) in RT-PCR, qPCR, and MSD analyses. Exact *p*-values for bars with asterisks are 1.77e-5, 1.46e-6, and 4.25e-7; 9.40e-5, 6.44e-7, and 3.66e-7; 1.33e-9, 9.47e-15, and 5.83e-11; 1.58e-5; 3.17e-12 and 1.20e-15; 0.0005, 2.73e-5, and 1.41e-5; 0.0004, 1.71e-6, and 5.56e-7. Source data are provided as a Source Data file.

To test the two *SCN1A* ASO hits in vivo, ASO-135 and ASO-136 were administered at four different doses (0.3, 1.25, 5, and 20 μg) to WT C57BL/6N neonatal mouse brains via a single intracerebroventricular (ICV) bolus injection at postnatal day 2. In addition to PBS as a mock control (−), 20 μg of a non-targeting (NT) ASO control was injected^30^. Five days post ICV injection, RNA and protein were extracted from coronal slices. RT-PCR analysis was performed to determine target engagement by measuring the percentage of non-productive mRNA as described above. Results showed that both ASO-135 and ASO-136 led to a dose-dependent decrease of non-productive EI (Fig. 6a, Supplementary Fig. 9a, b). This reduction translated to an inversely correlated dose-dependent increase in productive mRNA as measured by qPCR relative to the PBS control (Fig. 6b). The non-targeting ASO control does not alter splicing or mRNA levels compared to the PBS group (Fig. 6a, b, Supplementary Fig. 9a, b). Next, Na_V_1.1 protein was measured by Meso Scale Discovery (MSD) assay^36^ using validated detection and capture antibodies (Supplementary Fig. 5d, e, respectively). Results showed a dose-dependent upregulation of Na_V_1.1 protein for both targeting ASOs, while the non-targeting ASO control had no effect on protein levels relative to the PBS control group. These results indicate that the effect of TANGO ASOs in vitro translate to in vivo. Moreover, the titratable nature of TANGO ASO-mediated RNA and protein upregulation is also evident in vivo suggesting that by using this technology we could tightly control protein levels and reduce the risk of overexpression.

## Discussion

In this study, we present a technology called TANGO (targeted-augmentation of nuclear gene output) that is capable of upregulating endogenous protein expression by leveraging multiple types of non-productive (NMD-inducing) alternative splicing events. Using a bioinformatics pipeline, we have identified NMD-inducing AS events in ~1/3 of all protein-coding genes. We demonstrated the TANGO mechanism using four selected targets (*PCCA*, *SYNGAP1*, *CD274*, and *SCN1A*), and screening 144 ASOs targeting three different types of non-productive splicing events. The identified ASO hits showed a consistent mechanism of action indicative of TANGO, such that a reduction of the non-productive splicing event led to an inversely correlated increase of productive mRNA. Moreover, consistent with the mechanism of action of the hit ASOs, we observed that the level of ASO-mediated mRNA upregulation correlates with the abundance of the targeted NMD-inducing events (Supplementary Fig. 10, *R*^2^ = 0.9886, *p* = 0.0057). The increase in productive mRNA translated into an increase in full-length and fully functional protein regardless of the protein function. In addition, we showed that two FDA-approved chemistries (2′MOE and PMO) and various ASO lengths reduce non-productive splicing, upregulate productive mRNA, and increase protein expression. As these two chemistries have very different structures, pharmacokinetics, and biodistribution^8^, these results indicate that the TANGO approach can leverage diverse chemistries to potentially target a wide variety of organs and diseases.

Finally, we show that this approach is feasible in vivo by demonstrating that two active ASOs initially discovered in vitro, which are directed against a non-productive splicing event in *Scn1a*, increase both mRNA and protein in the brain of treated mice. Overall, the observed increase of productive mRNA and protein for the four selected targets is ASO-dose dependent indicating that the level of upregulation is highly titratable, thereby reducing the risk of overexpression. By achieving proof of concept with the four selected targets, we were able to demonstrate that TANGO can upregulate endogenous proteins of any size, type, or function. Moreover, as ASOs modulate splicing at the level of pre-mRNA, only expressed targets can be upregulated.

**Fig. 7:**
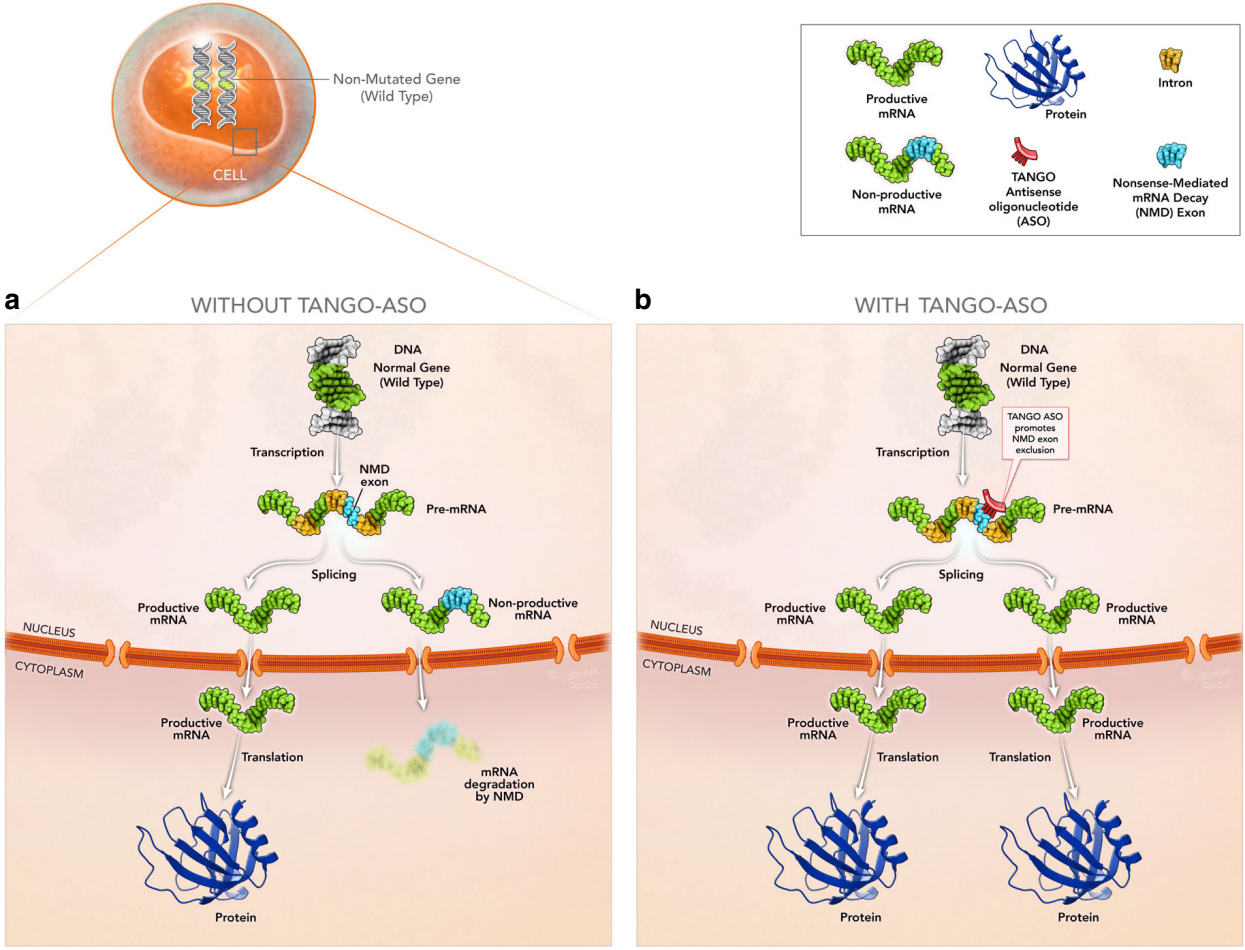
TANGO ASOs prevent NMD to increase productive mRNA and protein. **a** Example of a cell expressing a pre-mRNA that is alternatively spliced to generate a productive mRNA and a non-productive mRNA. While the non-productive mRNA is degraded by NMD, the productive mRNA is translated into protein. **b** Example of a cell treated with a TANGO ASO targeting the non-productive AS event (e.g. exon inclusion) and preventing the inclusion of the non-productive exon leading to an increase of the productive mRNA. The increased level of the productive mRNA results in increased protein production.

As TANGO exploits naturally occurring non-productive AS, we can employ this technology to upregulate protein expression of any gene with two wild-type alleles (Fig. 7), or a wild-type and a loss of function allele, or at least one hypomorphic allele. In one example, we speculate that the disease severity of *PCCA*-related propionic acidemia correlates with the level of PCCA residual function^37^; thereby upregulation of a partially functional PCCA protein could presumably lead to an improvement in disease presentation. *CD274*, while not a disease-causing gene, encodes for PD-L1, a protein involved in the immune response^38^, and its upregulation could potentially benefit patients with autoimmune diseases such as uveitis^39^. Finally, increasing protein expression could be especially beneficial for genes associated with autosomal dominant haploinsufficiency diseases, such as *SYNGAP1* (autosomal dominant mental retardation 5^40^) and *SCN1A* (Dravet syndrome^41^). TANGO ASOs can increase expression by leveraging the wild-type alleles to restore physiological levels of the deficient proteins.

Since non-productive splicing events are present in a large number of mRNAs, ASO therapeutics may be used to specifically increase endogenous protein production for many genes. As many diseases are caused by a decrease in protein levels, our approach may have clinical applications. ASOs have been approved for the treatment of several genetic diseases and have been delivered safely to a variety of tissues. The observed increased levels of protein in wild-type animals is meaningful. In patients with one loss of function allele, even a small increase of functional protein from the wild-type allele could be therapeutically beneficial. The TANGO technology offers a gene-specific approach to treat the underlying causes of severe genetic diseases by precisely upregulating protein expression in cells in which the target gene is naturally expressed.

## Methods

### Transcript database and labeling of NMD junctions

Annotated transcripts were downloaded from GENCODE (v. 28) and REFSEQ (via UCSC). We labeled each annotated exon–exon junction as “coding” or “NMD”. Junctions are labeled “NMD” if and only if that junction is exclusively found in transcripts labeled “nonsense_mediated_decay” (GENCODE) or “NR” (REFSEQ).

### RNA-seq sample quality control

Several tools were used to assess RNA-seq sample quality including bamtools (v2.5.1)^42^, fastqc (v0.11.8)^43^, and rseqc (v3.0.0)^44^. We excluded six samples from the CNS datasets due to high difference of duplicated reads in R1 and R2 of the same paired-end sample. All QC metrics are in Supplementary Data 1.

### RNA-seq sample processing

All raw RNA-seq data were downloaded from the Sequence Read Archive (https://www.ncbi.nlm.nih.gov/sra) with the following identifiers: SRP026048, SRP107937, SRP174668, and ERP003613. All RNA-seq samples were aligned to the hg38 genome and our combined transcript database using STAR v2.6.1b^45^ to generate splice junction counts. We used the following non-default parameters: --outFilterType BySJout, --alignSJoverhangMin 8, --outSAMattributes All, --outFilterMismatchNmax 999, --outFilterMultimapNmax 20, --alignEndsType EndToEnd, and --outSAMstrandField intronMotif.

### Identification and quantification of NMD splicing events

We ran all samples through SUPPA2^46^ (v2.3) to define annotated alternative splicing events. Different approaches were then used to label and quantify each type of alternative splicing, as described below.

### Exon inclusion and exon skipping

We parsed the “skipped exon” events from SUPPA to obtain the inclusion and skipping junctions for each event. If the skipping junction was labeled “NMD”, the event was labeled “ES_NMD”. If either of the inclusion junctions were labeled as “NMD”, the event was labeled “EI_NMD”. Otherwise, the event is labeled “cassette exon.” Inclusion and skipping junction counts were retrieved from the STAR output, and these counts were summed across all events sharing the same alternatively spliced exon. The final PSI for the inclusion was calculated as: $\Psi = \frac{{{\mathrm{sj}}_{{\mathrm{inc}}}}}{{{\mathrm{sj}}_{{\mathrm{inc}}} + 2 \cdot {\mathrm{sj}}_{{\mathrm{skip}}}}}$. For inclusion events, Ψ_EI_NMD_ = Ψ. For skipping events, Ψ_ES_NMD_ = 1 − Ψ.

### Alternative 3′ and 5′ splice sites (A3 and A5)

We parsed the A3 and A5 events from SUPPA to obtain the junctions corresponding to each alternative event. If either the long or short junction is labeled as “NMD”, we report an NMD event (A3_NMD for A3 and A5_NMD for A5). If both junctions report NMD we do not report it because there is likely complex splicing in that region. Splice junction counts were retrieved from the STAR output. We report PSI as $\Psi _{A\{ 3,5\} \_{\mathrm{NMD}}} = \frac{{{\mathrm{sj}}_{{\mathrm{NMD}}}}}{{{\mathrm{sj}}_{{\mathrm{NMD}}} + {\mathrm{sj}}_{{\mathrm{coding}}}}}$.

### Alternative intron events

We parsed the retained intron events from SUPPA to obtain the list of AI. The AI event is labeled NMD (AI_NMD) if the event junction is labeled NMD. To calculate PSI, we first estimate the expression level of the exon within which the AI is located by summing all junctions using its 3′ and 5′ splice sites (and all other parent exons containing the same AI event). Usage of the AI junction will then fall within the range $\left[ {0,\frac{{{\mathrm{sj}}_{3^\prime } + {\mathrm{sj}}_{5^\prime }}}{2}} \right]$, because full use of the AI junction—i.e. no alternative intron retention—results in similar counts for the exon junctions and the AI junction and full alternative intron retention results in 0 counts for the AI junction. To calculate Ψ, we normalize the junction counts and set the exon expression as the upper limit such that it will fall within the range [0,1]: $\Psi _{{\mathrm{AI}}} \sim \frac{{{\mathrm{min}}\left( {{\mathrm{sj}}_{{\mathrm{AI}}},\frac{{{\mathrm{sj}}_{3^\prime } + {\mathrm{sj}}_{5^\prime }}}{2}} \right)}}{{\frac{{{\mathrm{sj}}_{3^\prime } + {\mathrm{sj}}_{5^\prime }}}{2}}}$.

### Annotation of disease relevance

We downloaded gene-disease association data from Orphadata (http://www.orphadata.org), the publicly available data repository of Orphanet. We extended the annotations to cover all gene symbol aliases as provided by the NCBI gene_info file (ftp://ftp.ncbi.nih.gov/gene/DATA/GENE_INFO/Mammalia/Homo_sapiens.gene_info.gz).

### Conservation of NMD exon inclusion events

We downloaded the human PhastCons 100-way scores from UCSC (http://hgdownload.soe.ucsc.edu/goldenPath/hg38/phastCons100way/hg38.phastCons100way.bw). We then generated three mutually exclusive BED files: (1) exons that cause NMD upon inclusion, (2) exons that are alternatively spliced as determined by SUPPA2 and that do not cause NMD upon inclusion, and (3) protein-coding constitutive exons from both references (all protein-coding exons excluding all those found in (1) and (2)). To calculate the conservation for each exon, we used bedtools^47^ (v2.27.1) bigWigAverageOverBed to determine the average conservation over the spliced region. Density plots were generated using ggplot2^48^ (v3.1.0) and cowplot (v1.0.0).

### Treatment with CHX, cell culture, and transfections

To determine the abundance of the non-productive mRNAs, cells (HEK293: PCCA, SYNGAP1; Huh7: CD274; ReNCell VM: SCN1A) were incubated with 50 μg/ml of CHX (Cell Signaling Technology) dissolved in DMSO for 3 h.

For PCCA, HEK293 cells were grown in EMEM with 10% FBS and 1 × 10^5^ cells were seeded in 24-well plate and reverse-transfected with 80 nM ASOs for initial screening or 1, 5, and 25 nM of selected ASO using Lipofectamine RNAiMax reagent (Invitrogen) according to manufacturer’s instructions. Total RNA was extracted using RNeasy mini kit (Qiagen) 24 h post-transfection and cDNA was synthesized with ImProm-II reverse transcriptase (Promega). Total protein was extracted with RIPA buffer (Cell Signaling Technology) 48 h post transfection.

For SYNGAP1, HEK293 cells were grown in EMEM with 10% FBS and 7 × 10^5^ cells were seeded in 6-well plate and reverse-transfected with 30, 60, and 120 nM of antisense oligonucleotide (ASO) using Lipofectamine RNAiMax reagent (Invitrogen) according to manufacturer’s instructions. The medium including ASO-lipid complex was changed after 6 h of transfection. For initial screening of ASOs, 80 nM of each ASO was used for the reverse transfection. Total RNA was extracted using RNeasy mini kit (Qiagen) 24 h post-transfection and cDNA was synthesized with ImProm-II reverse transcriptase (Promega). Total protein was extracted with RIPA buffer (Cell Signaling Technology) 48 h post transfection.

For CD274, Huh7 cells were grown in DMEM with 10% FBS and 1 × 10^5^ cells were seeded in a 12-well plate and reverse transfected with 5, 20, or 80 nM ASO using Lipofectamine RNAiMAX (Invitrogen) according to manufacturer’s instructions. For RT-PCR analysis, cells were treated, as indicated, with 50 μg/mL of CHX (Cell Signaling Technology) in DMSO for 3 h 21 h post transfection. Total RNA was extracted using RNeasy mini kit (Qiagen) 24 h post-transfection and cDNA was synthesized with ImProm-II reverse transcriptase (Promega).

For SCN1A, ReNcell VM cells were grown in complete NSC medium containing 20 ng/mL of bFGF and EGF each on laminin coated flasks (2D culture) until reaching ~90% confluency. The cells were then detached by accutase treatment, washed with PBS and cultured in complete NSC medium in ultra-low attachment surface 24-well polystyrene plate with 3, 8, and 20 μM ASO for gymnotic (free) uptake. Total RNA was extracted using RNeasy mini kit (Qiagen) 72 h post-ASO addition to media and cDNA was synthesized with ImProm-II reverse transcriptase (Promega).

### ASO gymnotic (free) uptake by ReNcells VM

Prior to proceeding with the gymnotic uptake, ASO was dissolved in nuclease-free H_2_O and the concentration of ASO solution was determined by OD_260_ nm absorbance. ReNcells VM (EMD Millipore, SCC008) were grown in laminin coated flasks in complete NSC medium. Complete NSC medium was prepared by supplementing maintenance medium (Millipore, SCM005) with bFGF (EMD Millipore, GF003) and EGF (EMD Millipore, GF144), to a final concentration 20 ng/mL each. Cells were detached from the flask by incubation with accutase (Millipore, SCR005), resuspended in prewarmed maintenance medium and pelleted at 300 × *g* by centrifugation. The supernatant was removed by aspiration and the cells was resuspended in complete NSC medium to ~1 × 10^6^/mL. In all, 0.5 mL of cell suspension (~5 × 10^5^ cells) was added to each well of an Ultra-Low Attachment Costar 24 well plate (Corning, 3473) containing ASO. The cells were mixed gently with ASO and were incubated at 37 °C, 5% CO_2_ for 72 h before harvesting.

### Extraction of total RNA from cultured ReNells

Total RNA was extracted from cultured ReNcells utilizing the Qiagen RNeasy Kit (Qiagen, 74106). Cells grown in ultra-low attachment wells were carefully transferred into a 1.5-mL Eppendorf tube, pelleted by 300 × *g* centrifugation for 3 min and lysed with 300 µL of buffer RLT supplemented with 1% β-mercaptoethanol. Cell lysates were transferred to 1.5 mL Eppendorf tubes. An equal volume of 70% ethanol (300 µL) was added to each cell lysate and mixed. The mixture (600 µL) was passed through a RNeasy column by centrifugation at 14,000 × *g* for 1 min. Each column was washed sequentially with 700 µL of Buffer RW1 and 700 µL Buffer RPE. Each empty column was centrifuged again after the final wash for 1 min at top speed. RNA was eluted from the column by 40 µL of RNase-free water.

### qPCR and RT-PCR assays

For expression analysis of the productive mRNA, TaqMan qPCR (Thermo Fisher SC) was performed for PCCA (Hs01120555_m1), SYNGAP1 (Catalog# Hs00405348_m1), Mo-Scn1a (Mm00450583_mH), CD274 (Catalog# Hs00204257_m1), RPL32 (Hs00851655_g1), Mo-Gapdh (Mm99999915_g1), and SYBR green qPCR was performed for human CD274 with forward primer 5′-AATGTGACCAGCACACTGAG-3′ and reverse primer 5′-GAATGTCAGTGCTACACCAAGG-3′, and probe-based qPCR (custom-designed, IDT) was performed for Hu-SCN1A with forward primer 5′-TGGGTTACTCAGAACTTGGA-3′, reverse primer 5′-GCATTCACAACCACCCTC-3′, and probe 5′-/56-FAM/CAAATCTCT/ZEN/CAGGACACTAAGAGCTCTGAGAC/3IABkFQ/-3′.

For PCCA, PCR analysis to amplify the productive and non-productive mRNAs was performed with forward primer 5′-GACCCCTACAAGTCTTTTGGTTT-3′ and reverse primer 5′-ATCACTTCCTGGTTGGATGC-3′. The cycling conditions were 30 s at 95 °C for denaturation, 30 s at 60 °C for annealing, and 60 s at 72 °C for extension for 30 cycles. The PCR products were separated on a 5% polyacrylamide gel and quantified with Multi Gauge software Version 2.3.

For SYNGAP1, PCR analysis to amplify the productive and non-productive mRNAs was performed using forward primer 5′-GACCCTATCAAGTGCACAGC-3′ and reverse primer 5′-CTCCTGCATAAGCCCAAAGAG-3′. The cycling conditions were 30 s at 95 °C for denaturation, 30 s at 60 °C for annealing, and 60 s at 72 °C for extension for 32 cycles. The PCR products were separated on a 5% polyacrylamide gel and quantified with Multi Gauge software Version 2.3.

For CD274, PCR analysis to amplify the productive and non-productive mRNAs was performed using forward primer 5′-GTCATCTGGACAAGCAGTG-3′ and a reverse primer 5′-GGATGCCACATTTTTTCACATC-3′. The cycling conditions were 30 s at 95 °C for denaturation, 30 s at 55 °C for annealing, and 30 s at 72 °C for extension for 29 cycles. The PCR products were separated on a 5% polyacrylamide gel and quantified with Multi Gauge software Version 2.3.

For Hu-SCN1A, PCR analysis to amplify the productive and non-productive mRNAs was performed with forward primer 5′-ATTGTTGATGTTTCATTGGTCAGTTTAACA-3′ and reverse primer 5′-GAAGAAGGACCCAAAGATGATGAAAATA-3′. The cycling conditions were 30 s at 95 °C for denaturation, 30 s at 55 °C for annealing, and 75 s at 72 °C for extension for 28 cycles. For Mo-Scn1a, PCR analysis to amplify the productive and non-productive mRNAs was performed with forward primer 5′-CAGTTTAACAGCAAATGCCTTGGGTT-3′ and reverse primer 5′-AAGTACAAATACATGTACAGGCTTTCCTCATACTTA-3′. The cycling conditions were 30 s at 95 °C for denaturation, 30 s at 56 °C for annealing, and 75 s at 72 °C for extension for 28 cycles. For Mo-Gapdh, PCR was performed with forward primer 5′-AGGTCGGTGTGAACGGATTTG-3′ and reverse primer 5′-GGGGTCGTTGATGGCAACA-3′. The cycling conditions were 30 s at 95 °C for denaturation, 30 s at 56 °C for annealing, and 60 s at 72 °C for extension for 24 cycles. The PCR products were separated on a 5% polyacrylamide gel and quantified with Multi Gauge software Version 2.3.

For ACOT8, PCR analysis to amplify the productive and non-productive mRNAs was performed using forward primer 5′-CTCAACCGAATTGCTGCTC-3′ and a reverse primer 5′-CCAAGAAGGCATAGTCGG-3′. The cycling conditions were 30 s at 95 °C for denaturation, 30 s at 55 °C for annealing, and 30 s at 72 °C for extension for 35 cycles. The PCR products were separated on a 5% polyacrylamide gel and quantified with Multi Gauge software Version 2.3.

For MRGBP, PCR analysis to amplify the productive and non-productive mRNAs was performed using forward primer 5′-CCGAATCCAGAGAGGAAC-3′ and a reverse primer 5′-CACGTCTTCCTTCATCTCC-3′. The cycling conditions were 30 s at 95 °C for denaturation, 30 s at 55 °C for annealing, and 30 s at 72 °C for extension for 35 cycles. The PCR products were separated on a 5% polyacrylamide gel and quantified with Multi Gauge software Version 2.3.

For CCDC84, PCR analysis to amplify the productive and non-productive mRNAs was performed using forward primer 5′-CTCAGATCCGTGAGGTGG-3′ and a reverse primer 5′-CCTTTCCAGCTTCTAGGTG-3′. The cycling conditions were 30 s at 95 °C for denaturation, 30 s at 55 °C for annealing, and 30 s at 72 °C for extension for 35 cycles. The PCR products were separated on a 5% polyacrylamide gel and quantified with Multi Gauge software Version 2.3.

For TMEM208, PCR analysis to amplify the productive and non-productive mRNAs was performed using forward primer 5′-CCACTCTATGAGCTCGATGG-3′ and a reverse primer 5′-CGATGGCTGTCAGTAGGATC-3′. The cycling conditions were 30 s at 95 °C for denaturation, 30 s at 55 °C for annealing, and 30 s at 72 °C for extension for 35 cycles. The PCR products were separated on a 5% polyacrylamide gel and quantified with Multi Gauge software Version 2.3.

For GLYR1, PCR analysis to amplify the productive and non-productive mRNAs was performed using forward primer 5′-GGCAGAGTCCCGAGAAG-3′ and a reverse primer 5′-CTGGCTGGAGTGAAGACAC-3′. The cycling conditions were 30 s at 95 °C for denaturation, 30 s at 55 °C for annealing, and 30 s at 72 °C for extension for 35 cycles. The PCR products were separated on a 5% polyacrylamide gel and quantified with Multi Gauge software Version 2.3.

For PARG, PCR analysis to amplify the productive and non-productive mRNAs was performed using forward primer 5′-GAGAGACATTTACAGCATGCAC-3′ and a reverse primer 5′-GCTGAGCACAACCTTCC-3′. The cycling conditions were 30 s at 95 °C for denaturation, 30 s at 55 °C for annealing, and 30 s at 72 °C for extension for 35 cycles. The PCR products were separated on a 5% polyacrylamide gel and quantified with Multi Gauge software Version 2.3.

For C1orf52, PCR analysis to amplify the productive and non-productive mRNAs was performed using forward primer 5′-GACTGGGAGAGGCACG-3′ and a reverse primer 5′-GCCATGTCAAGCTCTGGAG-3′. The cycling conditions were 30 s at 95 °C for denaturation, 30 s at 55 °C for annealing, and 30 s at 72 °C for extension for 35 cycles. The PCR products were separated on a 5% polyacrylamide gel and quantified with Multi Gauge software Version 2.3.

For DHX9, PCR analysis to amplify the productive and non-productive mRNAs was performed using forward primer 5′-GGCTTCATGGAAACTGGAC-3′ and a reverse primer 5′-GTTGTCTGACAAGTGACAGG-3′. The cycling conditions were 30 s at 95 °C for denaturation, 30 s at 55 °C for annealing, and 30 s at 72 °C for extension for 35 cycles. The PCR products were separated on a 5% polyacrylamide gel and quantified with Multi Gauge software Version 2.3.

For EYA3, PCR analysis to amplify the productive and non-productive mRNAs was performed using forward primer 5′-CCCAACAGTAGTGATTGGCTC-3′ and a reverse primer 5′-CTTCCACATGTACCTGGTCAC-3′. The cycling conditions were 30 sec at 95 °C for denaturation, 30 sec at 55 °C for annealing, and 30 sec at 72 °C for extension for 35 cycles. The PCR products were separated on a 5% polyacrylamide gel and quantified with Multi Gauge software Version 2.3.

For TEP1, PCR analysis to amplify the productive and non-productive mRNAs was performed using forward primer 5′-GTGGCTGCTGGTAACAG-3′ and a reverse primer 5′-GAGGGCAACTTCGGAAGG-3′. The cycling conditions were 30 s at 95 °C for denaturation, 30 s at 55 °C for annealing, and 30 s at 72 °C for extension for 35 cycles. The PCR products were separated on a 5% polyacrylamide gel and quantified with Multi Gauge software Version 2.3.

All primers used in the study are in Data Supplementary 5.

### Western blotting

Protein extracts were quantified by colorimetric assay using Pierce BCA protein assay kit (ThermoFisher).

For PCCA, immunoblotting was carried out with 20 μg of lysate. Anti-PCCA primary antibody (Cat# ab187686, 1:1000 dilution, overnight at 4 °C) and anti-rabbit IgG Alexa Fluror488 secondary antibody (Cat# A32731, 1:5000, 1 h at RT) were purchased from Abcam and Invitrogen, respectively. Anti-Vinculin monoclonal primary antibody (Cat# MA5-11690, 1:500 dilution, 1 h at room temperature, Thermo Fisher) and ECL Plex Goat-α-Mouse IgG-Cy3 secondary antibody (Cat# PA43009V, 1:2000, 1 h at RT) were purchased from Invitrogen and GE Healthcare, respectively. Blots were scanned using a Typhoon RLA 9000 imager (General Electric). Densitometric analysis was carried out using Multi Gauge software Version 2.3. For antibody validation, the TriFECTa DsiRNA Kit for PCCA was purchased from IDT (Design ID# hs.Ri.PCCA.13).

For SynGAP, immunoblotting was carried out with 60 μg of lysate. Anti-SynGAP primary antibody (Cat# 5539, 1:1000, overnight at 4 °C) and secondary anti-rabbit HRP-conjugated (Cat# 7074, 1:5000, 1 h at RT) were purchased from Cell Signaling Technology. IRDye 800CW donkey anti-rabbit IgG secondary antibody (Cat# 926-32213, 1:15,000, 1 h at RT) were purchased from LI-COR. For Vinculin, 10 μg of lysate was used and blotted with Vinculin (Cat# MA5-11690, 1 μg/mL, overnight at 4 °C, Thermo Fisher Scientific). IRDye 680RD Donkey anti-mouse secondary antibody (Cat# 926-68072, 1:15,000, 1 h at RT) were purchased from LI-COR. Blots were scanned using Typhoon RLA 9000 imager (General Electric) or Odyssey CLx (LI-COR). Densitometric analysis was carried out using Multi Gauge software Version 2.3. For antibody validation, SYNGAP1 siRNA was purchased from Dharmacon (Cat# SO-2690002G).

### Flow cytometry

Five days post-transfection, cells were lifted from culture plates in FACS buffer (ThermoFisher Scientific). Cells were stained with APC-anti-PD-L1 (1:250, BioLegend, #329708). Data from 10,000–15,000 cells were collected on a Guava easyCyte 12HT (EMD Millipore) flow cytometer. FMO was used to determine the positive gate. PD-L1 antibody was validated via overexpression using cDNA from the PD-L1/TCR Activator Mammalian Expression Kit purchased from BPS Bioscience (Cat# 60610). cDNA and TCR activator were specially requested not to be mixed.

### Animals

C57BL/6NCrl mice from Charles River Laboratories, MA were used in this study. Mouse studies were performed under protocols approved by the Institutional Animal Care and Use Committee (IACUC) of Stoke Therapeutics, Inc and were in accordance with the NIH Guide for the Care and Use of Laboratory Animals. All mice were maintained on a 12:12-h light:dark cycle and had ad libitum access to food and water throughout the experiments. Male and female mice were used in all experiments.

### Single bolus ICV injection in neonate mice

Lyophilized ASO was reconstituted in 1x PBS (Thermo Fisher, 10010023) and the concentration of ASO solution was determined by OD_260_ nm absorbance. For dosing solutions, reconstituted ASO was diluted to the desired concentration in PBS with 0.01% (weight to volume) fast green dye (Sigma Aldrich, F7252-5G). PBS with 0.01% fast green dye was prepared as the vehicle control. For ICV injection in P2 mice, pups were immobilized by gently restraining them on a soft tissue padded surface with two fingers. A 33-gauge needle (Hamilton, 7803-05, 0.375-inch long, point style 4, 12° beveled) attached to a 5-μL microvolume syringe (Hamilton, 7634-01) was used for the injection. The coordinates of the injection were ~1 mm lateral from the sagittal suture and −2 mm ventral. In total, 2 μL of ASO or PBS was injected slowly into one cerebral lateral ventricle. Injected mice were quickly returned to the nest and observed daily for survival and signs of stress.

### Tissue dissection

Two coronal section of the mouse brain close to the injection site (indicated with a red dot) was cut with juxtaposed razor blades. The rostral coronal section (~1 mm thick, yellow shaded area) was used for total RNA extraction and for analysis for *Scn1a* gene expression. The caudal coronal section (~2 mm thick, blue shaded area) was used for preparation of total protein lysate and for analysis for Na_V_1.1 protein expression. The dissected tissues were snap frozen in liquid nitrogen and stored in a −80 °C freezer until analysis.

### Extraction of total RNA from brain tissue

Total RNA was extracted from brain tissue utilizing the Qiagen RNeasy Kit (Qiagen, 74106). Brain tissue (~30 mg) was mixed with 400 µL of Qiazol reagent (Qiagen, 79306) and ~0.1 mL of zirconium oxide beads (Next Advance, ZROB05 and ZROB10) in a sterile microcentrifuge 1.5 mL RINO® tube (Next Advence, TUBE1R5-S). Brain tissue was then homogenized in a Bullet Blender Storm Bead Mill tissue homogenizer (Next Advance, BBY24M) at 4 °C and speed setting 8, for 4 min. The homogenate was briefly centrifuged at 13,000 × *g* to precipitate insoluble debris. The supernatant (~300 μL) was transferred to a new tube and mixed with 400 µL of fresh Qiazol and 250 µL of chloroform. The mixture was vortexed vigorously for 15 s, incubated at RT for 2–3 min, and centrifuged at 13,000 × *g* for 15 min at 4 °C. In all, ~340 µL of the upper aqueous layer was transferred to a new tube. The supernatant was mixed with 1 vol (340 µL) of 70% ethanol, incubated at RT 10 min and passed through a RNeasy column by centrifugation at 13,000 × *g* for 1 min. The column was washed sequentially with 700 µL of Buffer RW1 and 700 µL of buffer RPE. Each empty column was centrifuged at full speed for 1 min after the last wash and dried at RT for 5 min. Total RNA was eluted from the column with 50 µL of RNase-free water. RNA concentration was determined by OD_260_ nm absorbance.

### cDNA synthesis

cDNA was synthesized using the ImProm-II reverse transcriptase kit (Promega, A3803). In all, 1 µg of the RNA template was mixed with 0.5 µg of Oligo dT in a total volume 11 µL. The mixture was incubated at 70 °C for 6 min and quickly chilled to 4 °C. Master mix (9 µL) containing MgCl_2_, dNTPs, and Improm-II reverse transcriptase was added to the RNA and Oligo dT mixture and the reaction was carried out as follows: 25 °C anneal for 5 min, 42 °C extend for 60 min, 70 °C heat-inactivate for 15 min, followed by 4 °C hold.

### RT-PCR

PCR reaction was prepared by mixing the following reagent in a 0.2-mL PCR tube: 1X AmpliTaq Gold 360 Master Mix (Applied Biosystems, 4396790), forward and reverse primers (0.4 μM each), cDNA template (1 µL), and nuclease-free H_2_O in a total volume of 25 µL. The PCR cycle conditions were: 95 °C for 9 min for 1 cycle, 95 °C for 30 s, 56 °C for 30 s, 72 °C for 75 s for 30 cycles (for *Scn1a*) or 95 °C for 30 s, 56 °C for 30 s, 72 °C for 60 s for 25 cycles (for *Gapdh*), 72 °C for 5 min. PCR products was separated on a 5% TBE polyacrylamide gel (Bio-rad, Criterion Precast Gel 3450049) by electrophoresis. The gel was stained with SYBR Safe Dye (1:10,000 dilution, Thermo Fisher, S33102) for 30 min and was scanned using a Typhoon 9500 laser scanner (GE Healthcare Life Sciences).

Primers for mouse *Scn1a* transcript (Exon 21 – Exon 24):

Forward primer: 5′-CAGTTTAACAGCAAATGCCTTGGGTT-3′

Reverse primer: 5′-AAGTACAAATACATGTACAGGCTTTCCTCATACTTA-3′

Primers for mouse *Gapdh*:

Forward primer: 5′-AGGTCGGTGTGAACGGATTTG-3′

Reverse primer: 5′-GGGGTCGTTGATGGCAACA-3′

Predicted molecular weights of the PCR products: for mouse, productive transcript (containing exon 21, 22, 23, and 24): 498 bp; non-productive transcript (containing exon 21, 21N, 22, 23, and 24): 562 bp.

### Probe-based qPCR

Probe-based qPCR was prepared by mixing the following reagents in each well of a 384-well plate (Applied Biosystems, 4309849): cDNA (1 µL), 1X Taqman Gene Expression Master Mix (Applied Biosystems, 4370074), 1X primers/probe mix, and nuclease-free H_2_O, to a total volume of 10 µL. The PCR and optical reading of the plate were carried on with a QuantStudio 5 thermocycler (Thermo Fisher). qPCR reactions were performed in triplicate for each sample. qPCR cycle conditions were: 50 °C for 2 min for 1 cycle, 95 °C for 10 min for 1 cycle, 95 °C for 15 s, and 60 °C for 1 min for 40 cycles. ΔCt was calculated by subtracting the average Ct (of three technical replicates) of the reference gene from the average Ct (of three technical replicates) of gene of interest for each sample. The ΔCt values were converted into ΔΔCt values by subtracting the average ΔCt value of control samples from the ΔCt of the test samples. The ΔΔCt were then converted into 2^−ΔΔCt^ for fold change of the gene expression.

### Total protein preparation and normalization

Brain tissue was homogenized in 1X Tris buffered saline, pH 7.4, containing 1% TX-100 (Sigma Aldrich, T8787-10ML), 0.5% Nonidet P-40 (Sigma Aldrich, 56741-250ML-F), 0.25% Na-deoxycholate (Sigma Aldrich, D6750-2G) and 1 mM EDTA (Ambion, AM9260G). Tissue was homogenized in lysis buffer with a Teflon-coated mortar and pestle (Thomas Scientific, 3431D88) for 30 strokes, by hand. The protein concentration of the tissue lysate was measured with the BCA assay (Pierce, 23227) following the manufacturer’s protocol. The protein concentration of the brain lysate was adjusted to 4 mg/mL with the RIPA buffer.

### Densitometry analysis

Scanned images were imported into the MultiGauge V2.3 software (Fujifilm, Japan). Areas of interest were selected with the rectangle selection tool. The optical densitometry of the selected areas was measured and exported as 16-bit grayscales. The grayscales were background subtracted and were related to their expression levels.

### Quantification of Na_V_1.1 protein in mouse brain

Quantification of Na_V_1.1 protein in mouse brain was performed with the Meso Scale Discovery (MSD) method. Multi-array 96 small spot GAM plates (MSD, L45MA-2) were blocked with 5% MSD blocker B (MSD, R93BB-2) prepared in TBS-T (1X Tris buffered saline, pH 7.4, containing 1% TX-100), 100 µL/well, under constant shaking for 1 h. MSD blocker B was tapped out from the GAM plates and the wells were coated with capture antibody (NeuroMab, 75-023, 0.95 mg/mL, dilute to 1:200 in 5% Block B), 25 µL/well, under constant shaking for 4 h. The capture antibody was tapped out from the GAM plates and the wells were washed 3x with TBS-T (150 µL/well). MSD standards were prepared by diluting adult mouse whole-brain lysate with liver lysate. Liver lysate was used as diluent since expression of *Scn1a* mRNA or Na_V_1.1 is undetectable in mouse liver. The protein concentration of all standards was kept at 4 mg/mL. In total, 25 µL of standard or sample (4 mg/mL) were added to GAM plate wells in duplicate. The plates were sealed and incubated at 4 °C under constant shaking. The contents were tapped out and wells were washed 3x with TBS-T (150 µL/well). 25 µL of detection antibody (Alomone, ASC-001, 0.6 mg/mL, dilute to 1:250 in 5% Block B) were added to each well and incubated at RT for 2 h under constant shaking. The detection antibody was tapped out and wells were washed 3x with TBS-T (150 µL/well). In all, 25 µL of sulfo-tagged anti-rabbit Ab (MSD, R32AB-1, 0.5 mg/mL, dilute to 1:250 in 5% Block B) were added to each well and incubated at RT for 1 h under constant shaking. The contents were tapped out and wells were washed 5x with TBS-T. In total, 150 µL of 1x MSD Read Buffer T (diluted in dH_2_O) were added to each well. The plates were read immediately with the Meso Quickplex SQ 120 machine. A serial dilution of adult mouse brain lysate containing Na_V_1.1 was used as standards for the assay. The MSD signals for each standard were read from duplicate wells, imported to GraphPad Prism 8.0 software, and fit to a non-linear, quadratic (second-order) polynomial regression curve. The concentration of Na_V_1.1 in each sample was interpolated using the standard curve.

### Ethical oversight

All animal experiments were conducted under protocols approved by the Institutional Animal Care and Use Committee (IACUC) of Stoke Therapeutics, Inc. and were in accordance with the National Research Council’s Guide for the Care and Use of Laboratory Animals.

### Statistical analysis

For comparison of means between two independent groups, two-tailed Student *t* test was performed. Statistical significance for all experiments was defined as *p* < 0.001. All analyses were performed using the GraphPad Prism 8.0 software.

### Reporting summary

Further information on research design is available in the Nature Research Reporting Summary linked to this article.

## Supplementary information

Supplementary Information

Reporting Summary

Description of Additional Supplementary Files

Supplementary Data 1

Supplementary Data 2

Supplementary Data 3

Supplementary Data 4

Supplementary Data 5

## Data Availability

The source data underlying Figs. 1, 2b, c, 3b, c, 4a–d, 5a–c, and 6a–c and Supplementary Figs. 1, 2b, c, 3a–e, 4a–e, 5, 6a, b, 7a–f, 8a, c, 9a, b, and 10 are provided as a Source Data file. All data are available from the corresponding author on reasonable requests. Source data are provided with this paper.

## Source data

Source Data
