# Supplementary material for: Antisense oligonucleotide modulation of non-productive alternative splicing upregulates gene expression
Source: Nat Commun. 2020 Jul 9;11:3501. doi: 10.1038/s41467-020-17093-9 (PMC7347940; doi:10.1038/s41467-020-17093-9)
Supplement: Supplementary file 1 — Supplementary Information [file 41467_2020_17093_MOESM1_ESM.pdf]

SUPPLEMENTARY INFORMATION

Antisense oligonucleotide modulation of non-productive alternative splicing upregulates gene expression

Lim et al.

Supplementary Figures

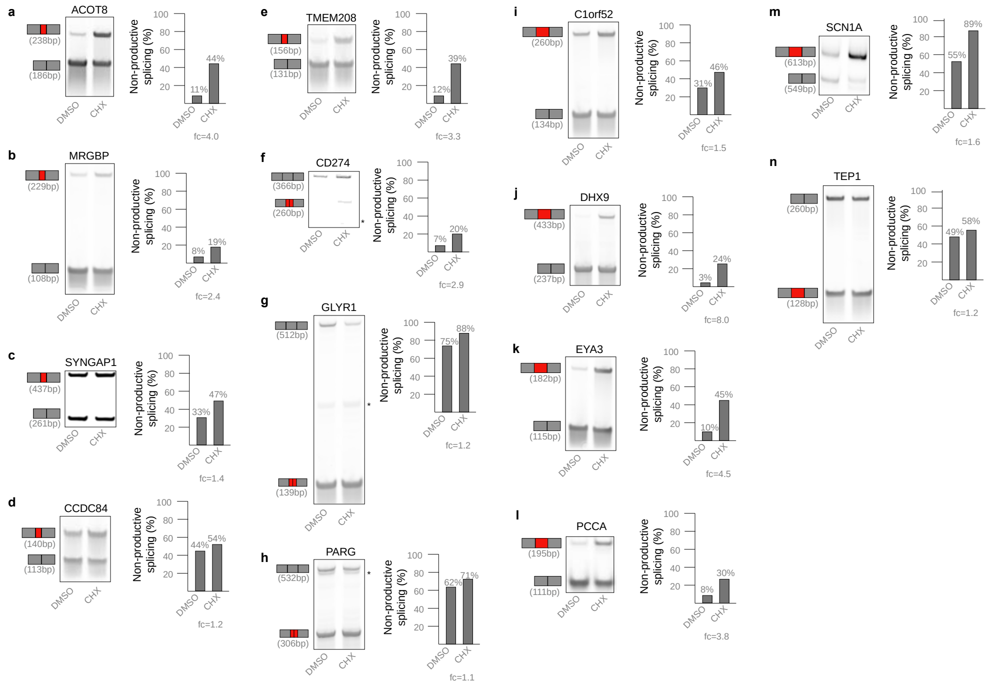

Supplementary Figure 1. Validation of different types of NMD events. RT-PCR and densitometric quantifications of RT-PCR validation in cells treated with 50ug/mL of CHX or equal volume of DMSO for 3h for (**a**) Alternative splice site in *ACOT8* in RenCell VM; (**b**) Alternative splice site in *MRGBP* in RenCell VM; (**c**) Alternative splice site in *SYNGAP1* in HEK293; (**d**) Alternative splice site in *CCDC84* in RenCell VM; (**e**) Alternative splice site in *TMEM208* in RenCell VM; (**f**) Alternative intron (exitron) in *CD274* in Huh7; (**g**) Alternative intron (exitron) in *GLYR1* in RenCell VM; (**h**) Alternative intron (exitron) in *PARG* in RenCell VM; (**i**) Alternative cassette exon in *C1orf52* in RenCell VM; (**j**) Alternative cassette exon in *DHX9* in RenCell VM; (**k**) Alternative cassette exon in *EYA3* in RenCell VM; (**l**) Alternative cassette exon in *PCCA* in HEK293; (**m**) Alternative cassette exon in *SCN1A* in RenCell VM; (**n**) Alternative cassette exon in *TEP1* in RenCell VM (bp denotes sizes of PCR products in base pairs, fc represents fold change of PCR products treated with CHX relative to DMSO). Red and grey rectangles denote NMD-inducing and protein-coding exons, respectively. Asterisk indicates additional alternative splicing events that were detected at a very low abundant level. The validation experiment of the 14 events shown in figure was done with n=1 sample over 1 experiment. Source data are provided as a Source Data file.

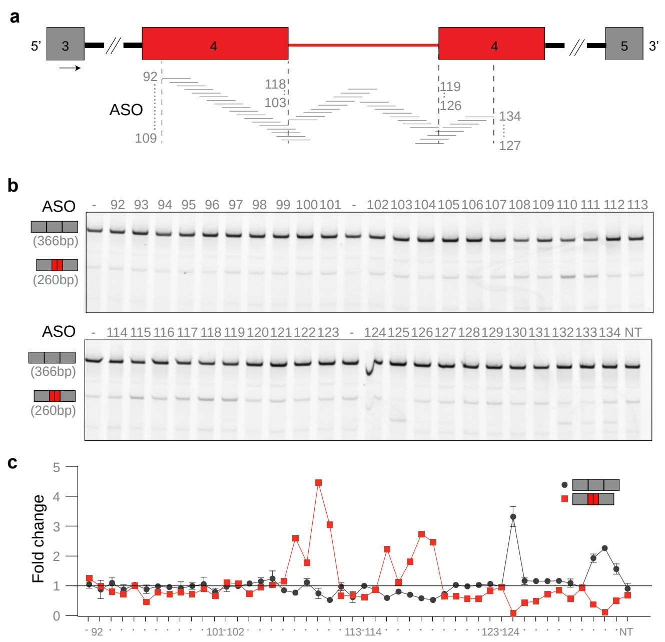

Supplementary Figure 2. ASO walk targeting the non-productive alternative intron event in *CD274*. (**a**) Diagram of ASO walk. 43 18-mer ASOs were designed to cover the non-productive alternative intron in *CD274* exon 4. Black lines denote introns, grey rectangles denote constitutive exons, and red rectangle denotes non-productive alternative intron. (**b**) A representative RT-PCR PAGE from Huh7 cells transfected with ASOs and a non-targeting ASO control at 80 nM concentration or no ASO control (-) for 24h. To ensure quantification of target engagement, cells were treated with 50ug/mL of CHX for 3h prior to harvesting. The bottom band (260bp) corresponds to the non-productive mRNA that results from the removal of the alternative intron within exon 4 and the top band (366bp) corresponds to the productive mRNA (n=2 biologically independent samples over 2 independent experiments). (**c**) Graph depicting the fold decrease of *CD274* non-productive mRNA based on densitometric quantification of RT-PCR products (red squares, with CHX, n=1) from panel (a) and the fold increase over no ASO control (-) of *CD274* productive mRNA determined by TaqMan qPCR (grey circles, without CHX, n=2). Data are presented as mean values +/- SD. Results show an inverse correlation between the reduction of non-productive mRNA and the increase of productive mRNA. ASO sequences are in Supplementary Data 4. Source data are provided as a Source Data file.

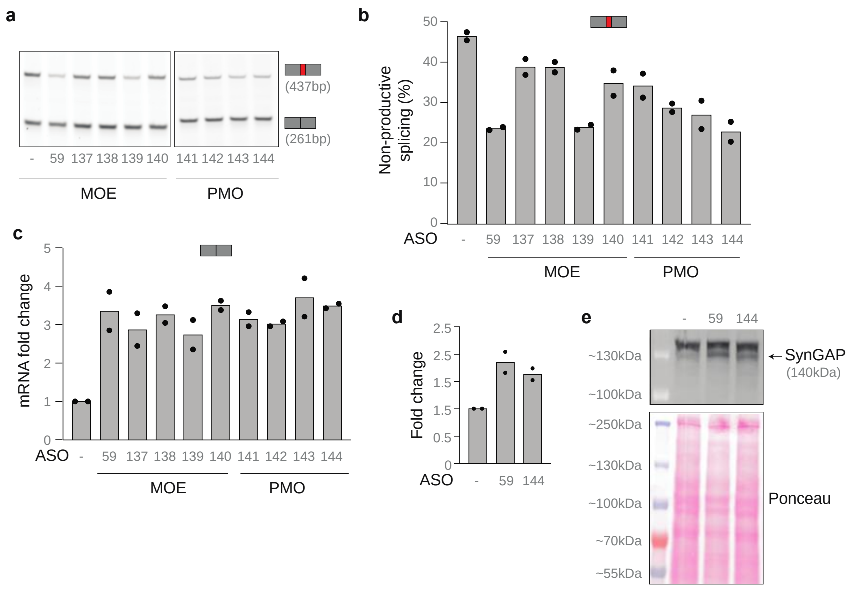

Supplementary Figure 3. 2'MOE-PS and PMO ASOs of various lengths reduce non-productive splicing events, upregulate mRNA, and increase protein. (**a**) Representative RT-PCR PAGE from MEF cells nucleofected with ASOs at 10 uM concentration or no-ASO control (-) for 24h. The top band (437bp) corresponds to the non-productive mRNA that results from the selection of the alternative 3'ss and the bottom band (261bp) corresponds to the productive mRNA that results from the canonical splicing of exon 11 (n=2 biologically independent samples over 2 independent experiment). (**b**) RT-PCR quantification (n=2). (**c**) qPCR of *SYNGAP1* ASOs targeting the alternative 3'ss event as in a and b (n=2). (**d**) Bar graph plotting the quantification of SynGAP of the (**e**) western blot analysis of MEF cells treated with an 18-mer 2'MOE-PS ASO or a 23-mer PMO or no-ASO control for 48h (n=2 biologically independent samples over 4 independent experiments). Equal protein loading was confirmed with Ponceau staining. ASO sequences are listed in Supplementary Data 4. Red and grey rectangles denote NMD-inducing and protein-coding exons, respectively. Source data are provided as a Source Data file.

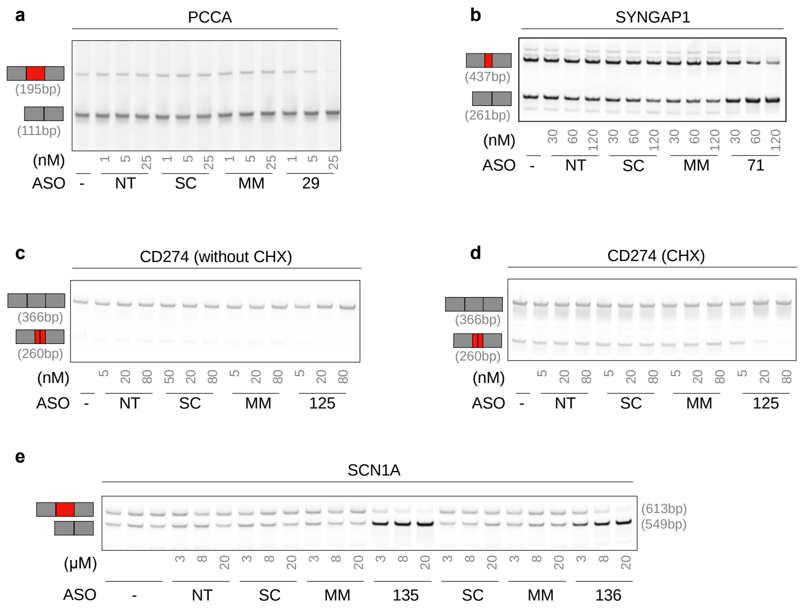

Supplementary Figure 4. ASOs reduce non-productive AS events and increase productive mRNA in a dose-dependent manner *in vitro*. Representative RT-PCR PAGE of cells transfected with 3 increasing concentrations of selected TANGO ASOs targeting (**a**) the exon inclusion event in *PCCA* in HEK293 (n=3 biologically independent samples over 2 independent experiments), (**b**) the alternative 3'ss in *SYNGAP1* in HEK293 (n=3 biologically independent samples over 2 independent experiments), (**c**) the alternative intron in *CD274* in Huh7 without CHX (n=3 biologically independent samples over 1 experiment), and **(d)** with 50ug/mL of CHX for 3h (n=3 biologically independent samples over 1 experiment), and (**e**) the exon inclusion event in *SCN1A* in ReNCell VM (n=3 biologically independent samples over 1 experiment). ASOs containing scrambled (SC) and mismatched (MM) sequences (see Supplementary Data 4) corresponding to each targeting ASO were included as controls. A non-targeting ASO control (NT) and no ASO control (-) were also included. Red and grey rectangles denote NMD-inducing and protein-coding exons, respectively. Source data are provided as a Source Data file.

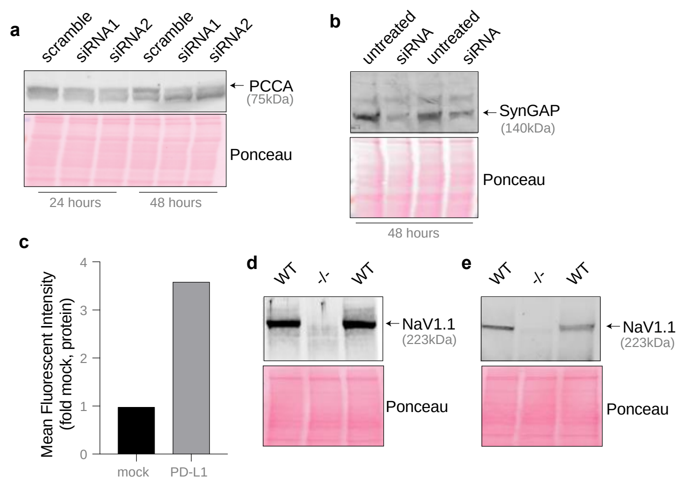

Supplementary Figure 5. Antibody validations. (**a**) Western blot of PCCA following treatment with 100nM siRNA against *PCCA* for 24h and 48h in HEK293 cells (n=1 sample over 1 experiment). (**b**) Western blot of SynGAP following treatment with 100nM siRNA against *SYNGAP1* for 48h in HEK293 cells (n=2 biologically independent samples over 1 experiment). **(c)** Flow cytometry derived fold change over control of the Mean Fluorescent Intensity after overexpressing PD-L1. (**d**) Specificity of the anti-Na_V1.1 capture antibody (n=1 knockout animal over 1 experiment) and (**e**) detection antibody was tested using total protein prepared from a *Scn1a* knock-out (-/-) mouse brain (middle lane) and brains of two WT littermates (left and right lanes) (n=1 knockout animal over 1 experiment). Na_V1.1 protein was not detected in brain lysate from the *Scn1a* knock-out mouse indicating that both antibodies are specifically detecting Na_V1.1. Source data are provided as a Source Data file.

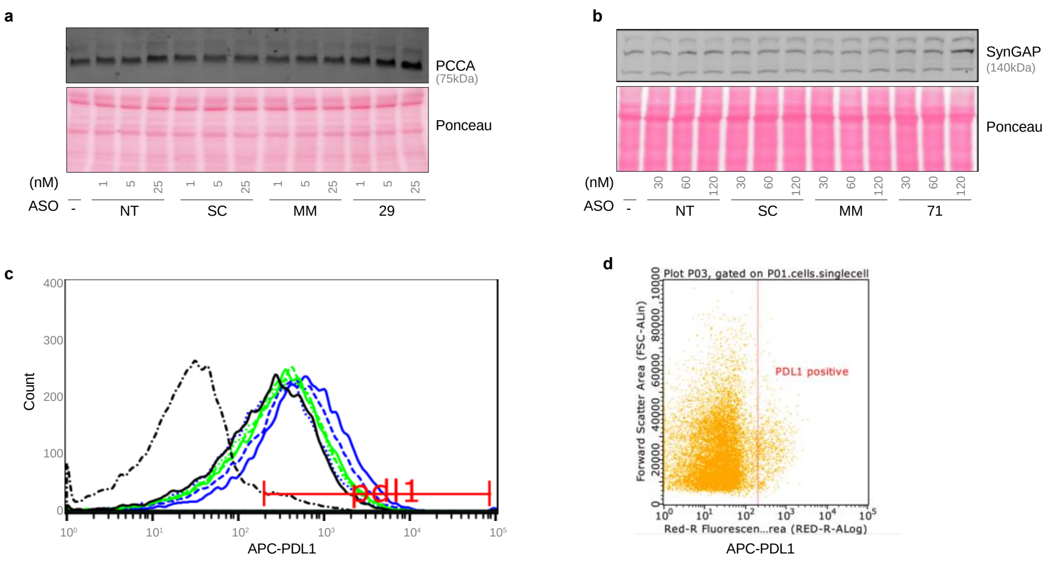

Supplementary Figure 6. TANGO ASOs increase protein levels in a dose-dependent manner *in vitro*. Representative western analysis of (**a**) PCCA (n=3 biologically independent sample over 2 independent experiments) and (**b**) SynGAP from HEK293 cells treated with the respective selected ASOs at 3 increasing concentrations for 48h (n=3 biologically independent sample over 2 independent experiments). Equal protein loading was confirmed with Ponceau staining. Fc = fold change over control. (**c**) Representative replicate of PD-L1 flow cytometry is shown. Unstained cells are represented by the black dash-dotted line. Mock transfected is represented by the solid black line. NT-ASO treated cells are plotted in green, while *CD274*-ASO treated cells are graphed in blue. For both NT-ASO and *CD274*-ASO treated cells, 5 nM is the dotted line, 20 nM is the dashed line, and 80 nM is the solid line. (**d**) The PD-L1 gate shows the cells included for analysis based off of unstained control. ASOs containing scrambled (SC) and mismatched (MM) sequences (see Supplementary Table 4) corresponding to each ASO were included as controls. A non-targeting ASO control (NT) and no ASO control (-) were also included. Source data are provided as a Source Data file.

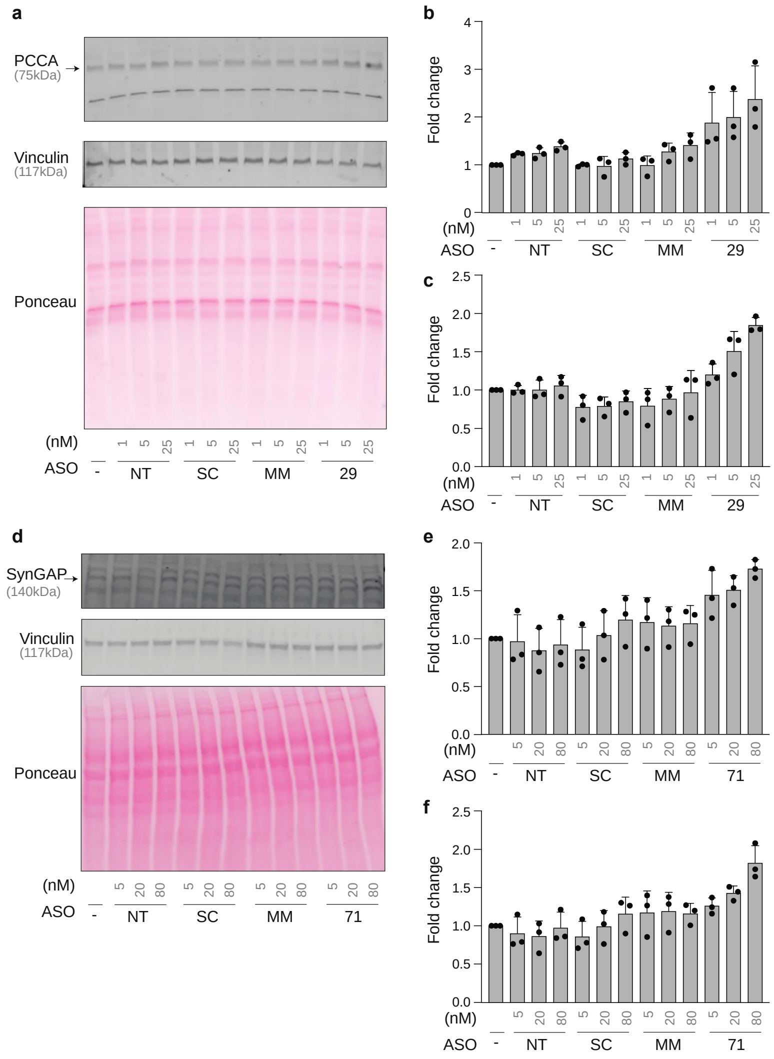

Supplementary Figure 7. Protein normalization using either an internal control protein or Ponceau led to similar results. Representative western analysis of (**a**) PCCA (n=3 biologically independent sample over 2 independent experiments) and (**d**) SynGAP from HEK293 cells treated with their corresponding ASO hits for 48h (n=3 biologically independent sample over 2 independent experiments). Equal protein loading was confirmed by Vinculin as internal control and with Ponceau staining. Bar graph plotting the quantification of PCCA or SynGAP protein normalized by (**b**) Vinculin or (**c**) Ponceau and (**e**) or (**f**) Ponceau, respectively. ASOs containing scrambled (SC) and mismatched (MM) sequences (see Supplementary Data 4) corresponding to each ASO were included as controls. A non-targeting ASO control (NT) and no ASO control (-) were also included. Data are presented as mean values +/- SD. Source data are provided as a Source Data file.

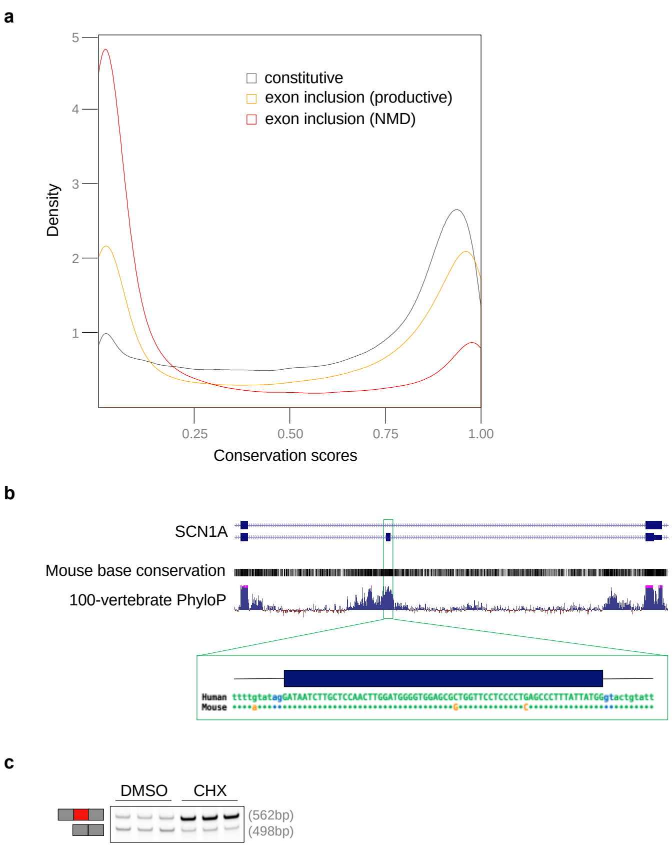

Supplementary Figure 8. Non-productive exon inclusion of 20x in *SCN1A* is conserved in mouse (21x in *Scn1a*). (**a**) PhastCons 100-way scores were used to compare conservation between constitutive and alternatively spliced exons in human (see Methods). Constitutive exons are defined as protein-coding exons from the human transcriptome database that are not alternatively spliced. Exon inclusion events derived from alternative splicing are categorized into productive (protein-coding) or non-productive (NMD). Each comparison of conservation scores is shown as a density plot. (**b**) Diagram of UCSC Genome Browser base conservation and 100-vertebrate PhyloP score tracks of *SCN1A* gene from exon 20 to exon 21 in human. Green box denotes the non-productive exon 20x in *SCN1A* and the sequence similarity between exon 20x in human and the non-productive exon 21x in mouse. (**c**) RT-PCR analysis of N2a mouse cells treated with CHX or DMSO detecting the non-productive exon inclusion event (562bp) and productive mRNA of *Scn1a (*n=3 biologically independent samples over 1 experiment). Red and grey rectangles denote NMD-inducing and protein-coding exons, respectively. Source data are provided as a Source Data file.

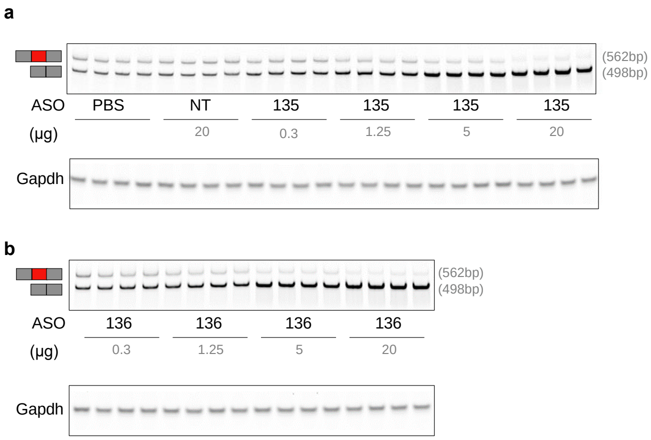

Supplementary Figure 9. Dose-dependent effects of ASO-135 and ASO-136 on expression of *Scn1a* mRNA in WT C57BL/6NCrl neonatal mouse brains. WT C57BL/6NCrl mice were ICV-injected at postnatal day 2 with either (**a**) PBS, a non-target control (NT, 20 µg), ASO-135 or ASO-136. ASO-135 and (**b**) ASO-136 were tested with 4 different doses (0.3, 1.25, 5 or 20 µg). Mice were euthanized 5 days after injection and a coronal section of the mouse brain was used for analysis of mRNA expression. TBE PAGE of RT-PCR products shows expression of *Scn1a* productive transcript (498bp) and non-productive, NMD-inducing transcript (562bp) in tested brain samples (n=4 biologically independent samples over 1 experiment). Red and grey rectangles denote NMD-inducing and protein-coding exons, respectively. Source data are provided as a Source Data file.

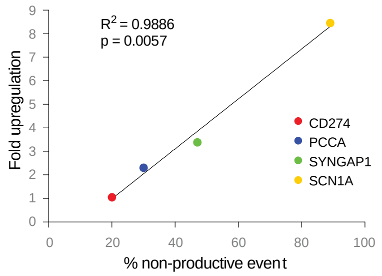

Supplementary Figure 10. Correlation between the abundance of CHX-treated non-productive AS event and the level of ASO-mediated upregulation of productive mRNA. The percentage of non-productive event of each target is calculated from the densitometric quantification of RT-PCR products in cells treated with 50ug/mL of CHX for 3h (Supplementary Fig. 2e-h). Fold upregulation for each target is based on the mean of qPCR analysis of the highest dose in the dose-dependent studies (n=3 for each target) (Fig. 4). ASOs used are ASO-29 (*PCCA*), ASO-71 (*SYNGAP1*), ASO-125 (*CD274*), and ASO-135 (*SCN1A*). R^2 measures the goodness-of-fit of linear regression. The p value is calculated from the *t* ratio by dividing the slop by its standard error. Source data are provided as a Source Data file.

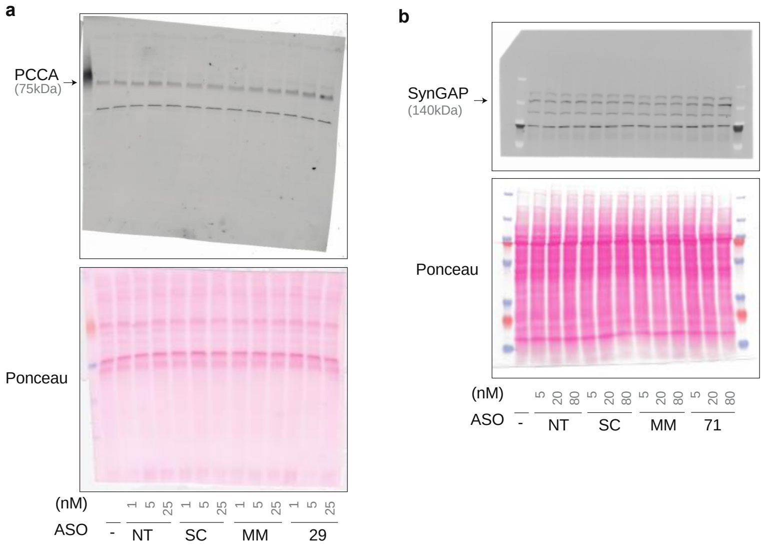

Supplementary Figure 11. Full-size image scans of corresponding Supplementary Figure 6a (PCCA) and 6b (SynGAP).
