# Supplementary material for: Antisense oligonucleotide modulation of non-productive alternative splicing upregulates gene expression
Source: Nat Commun. 2020 Jul 9;11:3501. doi: 10.1038/s41467-020-17093-9 (PMC7347940; doi:10.1038/s41467-020-17093-9)
Supplement: Supplementary file 2 — Reporting Summary [file 41467_2020_17093_MOESM2_ESM.pdf]

nature research

Corresponding author(s): Isabel Aznarez

Last updated by author(s): May 14, 2020

Reporting Summary

Nature Research wishes to improve the reproducibility of the work that we publish. This form provides structure for consistency and transparency in reporting. For further information on Nature Research policies, see our Editorial Policies and the Editorial Policy Checklist.

Statistics

For all statistical analyses, confirm that the following items are present in the figure legend, table legend, main text, or Methods section.

n/a	Confirmed	
☐	☒	The exact sample size (*n*) for each experimental group/condition, given as a discrete number and unit of measurement
☐	☒	A statement on whether measurements were taken from distinct samples or whether the same sample was measured repeatedly
☐	☒	The statistical test(s) used AND whether they are one- or two-sided<br>*Only common tests should be described solely by name; describe more complex techniques in the Methods section.*
☒	☐	A description of all covariates tested
☒	☐	A description of any assumptions or corrections, such as tests of normality and adjustment for multiple comparisons
☐	☒	A full description of the statistical parameters including central tendency (e.g. means) or other basic estimates (e.g. regression coefficient) AND variation (e.g. standard deviation) or associated estimates of uncertainty (e.g. confidence intervals)
☐	☒	For null hypothesis testing, the test statistic (e.g. *F*, *t*, *r*) with confidence intervals, effect sizes, degrees of freedom and *P* value noted<br>*Give P values as exact values whenever suitable.*
☒	☐	For Bayesian analysis, information on the choice of priors and Markov chain Monte Carlo settings
☒	☐	For hierarchical and complex designs, identification of the appropriate level for tests and full reporting of outcomes
☒	☐	Estimates of effect sizes (e.g. Cohen's *d*, Pearson's *r*), indicating how they were calculated

Our web collection on statistics for biologists contains articles on many of the points above.

Software and code

Policy information about availability of computer code

Data collection	No software was used in collecting data.
Data analysis	Quality control of the RNA-seq data was performed using bamtools (v2.5.1), fastqc (v0.11.8), and rseqc (v3.0.0). We used STAR v2.6.1b to align RNA-seq data to GENCODE v.28 and SUPPA2 (v2.3) to identify putative NMD-inducing alternative splicing events. Quantifications were done using custom code and described in Methods. Conservation calculations and visualizations were done using bedtools (2.27.1) and the R packages ggplot2 (v3.1.0) and cowplot (v1.0.0). All statistical analyses were performed using the GraphPad Prism 8.0 software.

For manuscripts utilizing custom algorithms or software that are central to the research but not yet described in published literature, software must be made available to editors and reviewers. We strongly encourage code deposition in a community repository (e.g. GitHub). See the Nature Research guidelines for submitting code & software for further information.

Data

Policy information about availability of data

All manuscripts must include a data availability statement. This statement should provide the following information, where applicable:

- Accession codes, unique identifiers, or web links for publicly available datasets
- A list of figures that have associated raw data
- A description of any restrictions on data availability

All raw RNA-seq data used to support the findings of this study are available publicly and can be downloaded from the Sequence Read Archive (https://www.ncbi.nlm.nih.gov/sra) with the following identifiers: SRP026048, SRP107937, SRP174668, and ERP003613. The source data underlying Figs 1, 2b-c, 3b-c, 4a-d, 5a-c, 6a-c and Supplementary Figs 1, 2b-c, 3a-e, 4a-e, 5, 6a-b, 7a-f, 8a, c, 9a-b, 10 are provided as a Source Data file. All other data are available from the corresponding author on reasonable request.

Field-specific reporting

Please select the one below that is the best fit for your research. If you are not sure, read the appropriate sections before making your selection.

- [x] Life sciences
- [ ] Behavioural & social sciences
- [ ] Ecological, evolutionary & environmental sciences

For a reference copy of the document with all sections, see nature.com/documents/nr-reporting-summary-flat.pdf

Life sciences study design

All studies must disclose on these points even when the disclosure is negative.

Sample size	There were 4 to 9 animals in each experiment group. The sample size was calculated assuming a 2-fold increase in gene expression in the experimental groups and the standard deviation being 0.5-fold of gene expression. If the targeted significant level (alpha value) is 0.05 and the power of statistics (two tailed test) is 0.8, the minimum sample size for each group would be 4.
Data exclusions	No data was excluded from the analysis.
Replication	The in-life study was performed once with 4 to 9 animals in each experiment group.
Randomization	No randomization was performed. Dam mice were randomly assigned to different experimental groups. Neonatal mice born from the same dam received the same treatment to avoid using traumatizing methods for animal identification (tail / finger clipping, tattooing, etc.) in the neonatal mice.
Blinding	Blinding was not performed. The final results and conclusion of this study were drew completely from the outcomes of qPCR and MSD assays, which were performed using the same conditions for all the samples. Subjective observation or judgment from the researcher was not involved in this study.

Reporting for specific materials, systems and methods

We require information from authors about some types of materials, experimental systems and methods used in many studies. Here, indicate whether each material, system or method listed is relevant to your study. If you are not sure if a list item applies to your research, read the appropriate section before selecting a response.

Materials & experimental systems

n/a	Involved in the study
[ ]	[x] Antibodies
[ ]	[x] Eukaryotic cell lines
[x]	[ ] Palaeontology and archaeology
[ ]	[x] Animals and other organisms
[x]	[ ] Human research participants
[x]	[ ] Clinical data
[x]	[ ] Dual use research of concern

Methods

n/a	Involved in the study
[x]	[ ] ChIP-seq
[ ]	[x] Flow cytometry
[x]	[ ] MRI-based neuroimaging

Antibodies

Antibodies used	Anti-PCCA primary antibody (Cat# ab187686) from Abcam<br>Anti-rabbit IgG Alexa Fluror488 secondary antibody (Cat# A32731) from Invitrogen and (ab150077) from Abcam<br>Anti-Vinculin monoclonal primary antibody (Cat# MA5-11690) from Invitrogen<br>ECL Plex Goat-α-Mouse IgG-Cy3 secondary antibody (Cat# PA43009V) from GE Healthcare.<br>Anti-SynGAP primary antibody (Cat# 5539) from Cell Signaling Technology<br>Secondary anti-rabbit HRP-conjugated (Cat# 7074) from Cell Signaling Technology<br>IRDye 800CW donkey anti-rabbit IgG secondary antibody (Cat# 926-32213) from LI-COR<br>Anti-NaV1.1 detection antibody (Cat# ASC-001) from Alomone, lot number: ASC001AN2802<br>Anti-NaV1.1 capture antibody: anti-NaV1.1 (Cat# 75-023) from NeuroMab, lot number: 413-7RR-87f<br>Goat anti-rabbit Antibody, SULFO-TAG Labeled (Cat# R32AB-1) from Meso Scale Diagnostics<br>PD-L1: APC-anti-PD-L1 (#329708) from BioLegend
Validation	Antibody validations were performed as follows:<br><br>Western blot of PCCA following treatment with 100nM siRNA against PCCA for 24h and 48h in HEK293 cells<br><br>Western blot of SynGAP following treatment with 100nM siRNA against SYNGAP1 for 48h in HEK293 cells

Flow cytometry derived fold change of the Mean Fluorescent Intensity of cell overexpressing PD-L1 over control

Western blot of NaV1.1 from total protein prepared from a Scn1aknock-out (-/-) mouse brain and brains from two WT littermates using the capture and detection antibodies.

Manufacturers used the following controls:

PCCA ab187686 – Western blot positive controls: HeLa whole cell lysate, HEK293T whole cell lysate, Jurkat whole cell lysate, TCMK1 whole cell lysate, NIH 3T3 whole cell lysate.

SYNGAP1 #5539 – Western blot was confirmed with rat and mouse brain lysate and is predicted to cross react with human as there is 100% sequence homology with the epitope.

PD-L1 Biolegend 329708 – human peripheral blood lymphocytes were used a positive control.

Scn1a NeuroMab, 75-023 - Not shown on manufacturer's website.

Scn1a Alomone, ASC-001 – Mouse parietal cortex was used as a positive control. Rat brain lysate preincubated with negative control antigen was used to show specificity.

Eukaryotic cell lines

Policy information about cell lines

Cell line source(s)	HEK293 from ATCC, ReNCell VM from Millipore, Huh7 from JCRB cell bank
Authentication	None of the cell lines were authenticated.
Mycoplasma contamination	All cell lines tested negative for mycoplasma.
Commonly misidentified lines (See ICLAC register)	The cell lines used are not on the list.

Animals and other organisms

Policy information about studies involving animals; ARRIVE guidelines recommended for reporting animal research

Laboratory animals	Mus musculus, C57BL/6NCrl, both male and females. ICV injection was performed at postnatal day 2. Mice were euthanized at postnatal day 7 and brain tissues were harvested for analysis.
Wild animals	The study did not involve wild animals
Field-collected samples	The study did not involve field-collected samples.
Ethics oversight	All animal experiments were conducted under protocols approved by the Institutional Animal Care and Use Committee (IACUC) of Stoke Therapeutics Inc. and were in accordance with the National Research Council's Guide for the Care and Use of Laboratory Animals.

Note that full information on the approval of the study protocol must also be provided in the manuscript.

Flow Cytometry

Plots

Confirm that:

- [x] The axis labels state the marker and fluorochrome used (e.g. CD4-FITC).
- [x] The axis scales are clearly visible. Include numbers along axes only for bottom left plot of group (a 'group' is an analysis of identical markers).
- [x] All plots are contour plots with outliers or pseudocolor plots.
- [x] A numerical value for number of cells or percentage (with statistics) is provided.

Methodology

Sample preparation	Five days post-transfection, cells were lifted from culture plates in FACS buffer (ThermoFisher Scientific). Cells were stained with APC-anti-PD-L1 (1:250, BioLegend, #329708). Data from 10,000-15,000 cells were collected on a Guava easyCyte 12HT (EMD Millipore) flow cytometer.
Instrument	Guava easyCyte 12HT Benchtop Flow Cytometer
Software	InCyte v3.2

Cell population abundance	N/A. We are using the cell line as a whole population; we are not subdividing the cell line.
Gating strategy	FSC/SSC to identify cells; SSC-A by SSC-B to identify single cells; unstained control to determine positive gating.

☒ Tick this box to confirm that a figure exemplifying the gating strategy is provided in the Supplementary Information.
