# Supplementary material for: Antisense oligonucleotide modulation of non-productive alternative splicing upregulates gene expression
Source: Nat Commun. 2020 Jul 9;11:3501. doi: 10.1038/s41467-020-17093-9 (PMC7347940; doi:10.1038/s41467-020-17093-9)
Supplement: Supplementary file 3 — Description of Additional Supplementary Files [file 41467_2020_17093_MOESM3_ESM.pdf]

Description of Additional Supplementary Files

File Name: Supplementary Data 1
Description: RNA-seq data table and QC metrics.

File Name: Supplementary Data 2
Description: NMD events from RNA-seq analysis.

File Name: Supplementary Data 3
Description: NMD events from RNA-seq analysis used for CHX validations.

File Name: Supplementary Data 4
Description: ASO sequences.

File Name: Supplementary Data 5
Description: Primers used in this study.
